# Menin enhances c-Myc-mediated transcription to promote cancer progression

**DOI:** 10.1038/ncomms15278

**Published:** 2017-05-05

**Authors:** Gongwei Wu, Mengqiu Yuan, Shengqi Shen, Xiaoyu Ma, Jingwen Fang, Lianbang Zhu, Linchong Sun, Zhaoji Liu, Xiaoping He, De Huang, Tingting Li, Chenchen Li, Jun Wu, Xin Hu, Zhaoyong Li, Libing Song, Kun Qu, Huafeng Zhang, Ping Gao

**Affiliations:** 1 CAS Key Laboratory of Innate Immunity and Chronic Disease, Innovation Center for Cell Signaling Network, School of Life Science, University of Science and Technology of China, Hefei, Anhui 230027, China; 2 State Key Laboratory of Oncology in Southern China and Department of Experimental Research, Sun Yat-sen University Cancer Center, Guangzhou, Guangdong 510060, China

## Abstract

Menin is an enigmatic protein that displays unique ability to either suppress or promote tumorigenesis in a context-dependent manner. The role for Menin to promote oncogenic functions has been largely attributed to its essential role in forming the MLL methyltransferase complex, which mediates H3K4me3. Here, we identify an unexpected role of Menin in enhancing the transactivity of oncogene MYC in a way independent of H3K4me3 activity. Intriguingly, we find that Menin interacts directly with the TAD domain of MYC and co-localizes with MYC to E-Box to enhance the transcription of MYC target genes in a P-TEFb-dependent manner. We further demonstrate that, by transcriptionally promoting the expression of MYC target genes in cancer cells, Menin stimulates cell proliferation and cellular metabolism both *in vitro* and *in vivo*. Our results uncover a previously unappreciated mechanism by which Menin functions as an oncogenic regulatory factor that is critical for MYC-mediated gene transcription.

## Abstract (web summary)

Menin is a protein with context-dependent oncogenic or oncosuppressive roles; the oncogenic activity is mainly due to its function as a cofactor of the MLL1 histone methyltransferase complex. Here the authors show that Menin regulates c-Myc-dependent transformation independently of the MLL complex.

C-Myc (hereafter referred to as MYC) is an important oncoprotein whose deregulated expression has contributed to 30–50% of human malignancies^1^,^2^,^3^,^4^. As a transcription factor, MYC is critical for cell proliferation, apoptosis, differentiation, metabolism, somatic cell reprogramming and other key processes under normal or pathological conditions via regulation of numerous target genes^2^,^3^,^5^,^6^,^7^. It is known that MYC regulates up to 15% of all human genes, mostly by binding to their canonical E-box regions to promote transcription^8^,^9^. Intriguingly, recent studies suggest that, instead of regulating transcription of new gene set, deregulated MYC amplifies the expression of extant active genes in a context-dependent manner, placing MYC as a master regulator in a new paradigm^10^,^11^,^12^. More recently, two groups provide new evidence to suggest that oncogenic MYC regulates gene expression selectively to promote cellular growth and tumour progression^13^,^14^. Hence, it remains an area of active investigation as to how MYC regulates gene transcription under normal or pathological circumstances, especially in oncogenesis.

Menin is an enigmatic protein whose emerging roles in cancer development have attracted a lot of attentions recently^15^,^16^. Menin displays the unique ability to either suppress tumorigenesis in the endocrine lineage or promote oncogenic functions in the liver and haematopoietic lineage^17^,^18^,^19^,^20^,^21^,^22^,^23^,^24^. However, its molecular mechanism of action has not been clearly defined. Menin was originally identified as a predominantly nuclear protein encoded by the Multiple Endocrine Neoplasia type 1 (*MEN1*) gene, whose germline mutation leads to tumours of endocrine pancreas, pituitary and parathyroid^21^,^22^,^23^,^24^. Interestingly, the tumour suppressive role of Menin is cell type-specific, e.g., disruption of Menin in the liver or haematopoietic system does not result in tumours^17^,^18^,^19^,^20^. In contrast, Menin that is aberrantly expressed in hepatocellular carcinoma (HCC) promotes liver tumorigenesis partially by regulating the expression of Yes-associated protein (Yap1)^18^, prompting a paradoxically oncogenic role for a tumour suppressor. It has been suggested that Menin promotes oncogenic function at least in part by serving as an essential oncogenic cofactor for the mixed lineage leukemia (MLL) gene products MLL1 and MLL2, which are SET domain-containing histone methyltransferases (HMTs), mediating trimethylation of histone H3 at lysine 4 (H3K4me3)^19^,^25^,^26^. H3K4me3 is observed predominantly at transcriptionally active loci, which has been linked to transcription initiation^27^,^28^,^29^,^30^. However, emerging evidence suggests transcriptional regulatory roles for Menin independent of the MLL methyltransferase complex^31^,^32^,^33^.

As aforementioned lines of evidence suggest that the multifaceted regulatory functions for both MYC and Menin are context dependent, we reason that elucidation of these context-dependent regulations are critical to the understanding of these two proteins’ roles in tumorigenesis. Obviously, while it is well established that MYC binds with MAX and other cofactors to form a regulatory complex at the E-Box to initiate gene transcription^1^,^34^, it stands to reason that this MYC-mediated process is still dependent on other key factors that need yet to be identified. Intriguingly, our early analysis showed an extensive overlap between MYC- and Menin-regulated genes. These early observations prompt us to address whether and how Menin is involved in tumorigenesis in the context of MYC deregulation. We report here that Menin plays an important role in MYC-mediated transcriptional activities through P-TEFb, indicating that Menin is a potential accomplice of MYC during oncogenesis. Our findings suggest that combined targeting of MYC and Menin signalling might serve as an effective therapeutic strategy in cancer cells with aberrant expression of these oncoproteins.

## Results

### Menin upregulates the expression of MYC target genes

**Figure 1:**
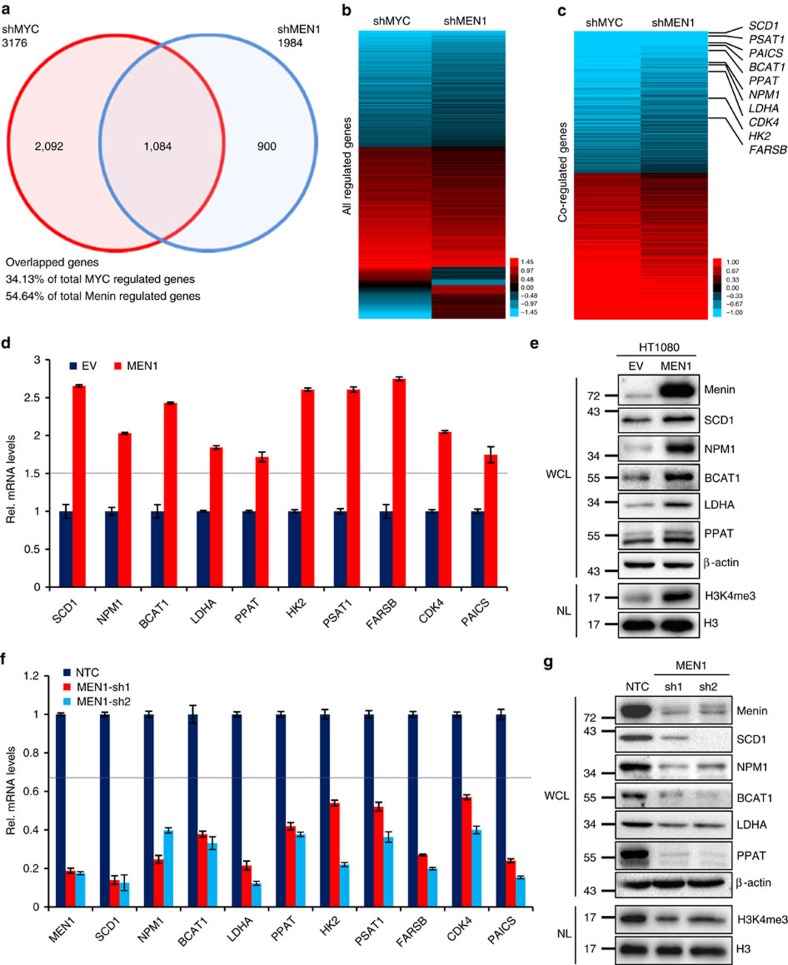
Menin upregulates the expression of MYC target genes. (**a**) Venn diagram of the RNA-seq showing the overlap of genes regulated by MYC and Menin. The statistical significance between MYC-regulated genes and Menin-regulated genes was determined using a Fisher’s exact probability, *P*<0.001. (**b**,**c**) Heat map showing all regulated genes (**b**) and co-regulated genes (**c**) by MYC or Menin (fold change >1.5). The significance of correlation between MYC-regulated genes and Menin-regulated genes was determined using a Spearman test, *P*<0.001. (**d**,**f**) Quantitative real-time PCR (qRT–PCR) analysing the mRNA levels of 10 MYC target genes (showing in **c**) in HT1080 cells stably expressing empty vector (EV) or Menin (**d**) and non-targeting control (NTC) or sh*MEN1* (**f**). Data were presented as mean (±s.d.) of three independent experiments. (**e**,**g**) Western blotting assay for the levels of Menin, MYC target genes and H3K4me3 in HT1080 cells stably expressing EV or Menin (**e**) and NTC or sh*MEN1* (**g**). β-Actin and H3 serve as loading controls. WCL, whole-cell lysate; NL: nuclear lysate.

MYC is a master regulator of transcription in proliferating cells. Genome-wide analysis studies have revealed that Menin also play broad roles in transcriptional regulation^18^,^31^,^35^,^36^. To investigate whether there are any correlations between MYC- and Menin-regulated gene expression, we comparatively analysed the gene profiling of human fibrosarcoma HT1080 cells with or without MYC or Menin (Fig. 1a–c). Our RNA-seq data showed that 3,176 genes were regulated by MYC while 1,984 genes were regulated by Menin with changes over 1.5-fold. Among them, 1,084 genes were simultaneously regulated by both MYC and Menin, which account for 34.13% of total MYC regulated genes and 54.64% of Menin, respectively, highlighting a high degree of overlap between MYC and Menin regulatory network (Fig. 1a–c). Gene ontology (GO) term enrichment analysis demonstrated that, consistent with the previously reported results, cell cycle-related genes as well as many metabolism-related genes were most frequently regulated by MYC, which was similar with the case of Menin, further suggesting the close correlation between MYC and Menin (Supplementary Fig. 1a).

To validate our RNA-seq data, we analysed 10 target genes that were upregulated by both MYC and Menin (Fig. 1c). Quantitive real time PCR (qRT–PCR) analysis confirmed the regulation of these genes by MYC in HT1080 cells with MYC overexpression or knockdown (Supplementary Fig. 1c,e). Western blotting assay with available antibodies against SCD1, NPM1, BCAT1, LDHA and PPAT also confirmed that their protein levels were regulated by MYC in both HT1080 and tet-inducible P493-6 B cells (Supplementary Fig. 1b,d,f). Consistent with RNA-seq results, mRNA levels of these MYC-regulated genes were all upregulated in HT1080 cells stably overexpressing Menin (Fig. 1d) and downregulated in HT1080 cells expressing shRNAs targeting Menin (Fig. 1f). Western blotting analysis revealed that protein levels of SCD1, NPM1, BCAT1, LDHA and PPAT were increased by overexpression of Menin (Fig. 1e) and decreased by Menin knockdown with shRNAs (Fig. 1g) in HT1080 cells. Similar results were also observed in HepG2 liver cancer cells expressing shRNAs targeting Menin (Supplementary Fig. 1g,h). Of note, our qRT–PCR analysis also confirmed that Menin did not affect the mRNA expression of *MYC*, *MAX* and some control genes whose mRNA expression was not alterred by MYC or Menin in RNA-seq results (Supplementary Fig. 1i,j). Moreover, an additional shRNA targeting the 3′UTR sequence of *MEN1* mRNA (sh3UTR) also decreased the expression of MYC target genes, which was recovered by restoring the expression of Menin (Supplementary Fig. 1k), confirming that the inhibition of MYC target gene expression by sh*MEN1* was not due to off-target effects of shRNAs. Taken together, our data showed that there was a significant correlation between Menin and MYC in regulation of gene expression, with Menin enhancing transcription of MYC target genes.

Menin is a non-methyl-transferase component of MLL HMT complex that mediates H3K4me3, which is usually associated with gene transcription initiation^30^,^37^. Apart from Menin, the H3K4me3 HMT complex has other three conserved trimethyltransferase factors, ASH2L, WDR5 and RBBP5 (refs 26, 37). Our results confirmed that H3K4me3 modification was indeed enhanced by Menin overexpression (Fig. 1e) and decreased by Menin knockdown (Fig. 1g) in HT1080 cells. To elucidate if H3K4me3 activity was involved in Menin-enhanced MYC target gene transcription, we performed gene knockdown experiments in HT1080 cells with shRNAs specifically targeting ASH2L-RBBP5, a minimized human heterodimer that activates the histone methyltransferases^38^. As expected, H3K4me3 modification was decreased when ASH2L was knocked down by shRNAs (Supplementary Fig. 2b). However, neither mRNA nor protein levels of MYC regulated genes were significanly affected by ASH2L shRNAs in HT1080 cells (Supplementary Fig. 2a,b). Similar results were observed in HT1080 cells expressing shRNAs targeting RBBP5 (Supplementary Fig. 2c,d), suggesting that enhanced transcription of MYC target genes by Menin was independent of the integrity of H3K4me3 HMT complex.

### Menin binds to E-box through interacting with MYC

**Figure 2:**
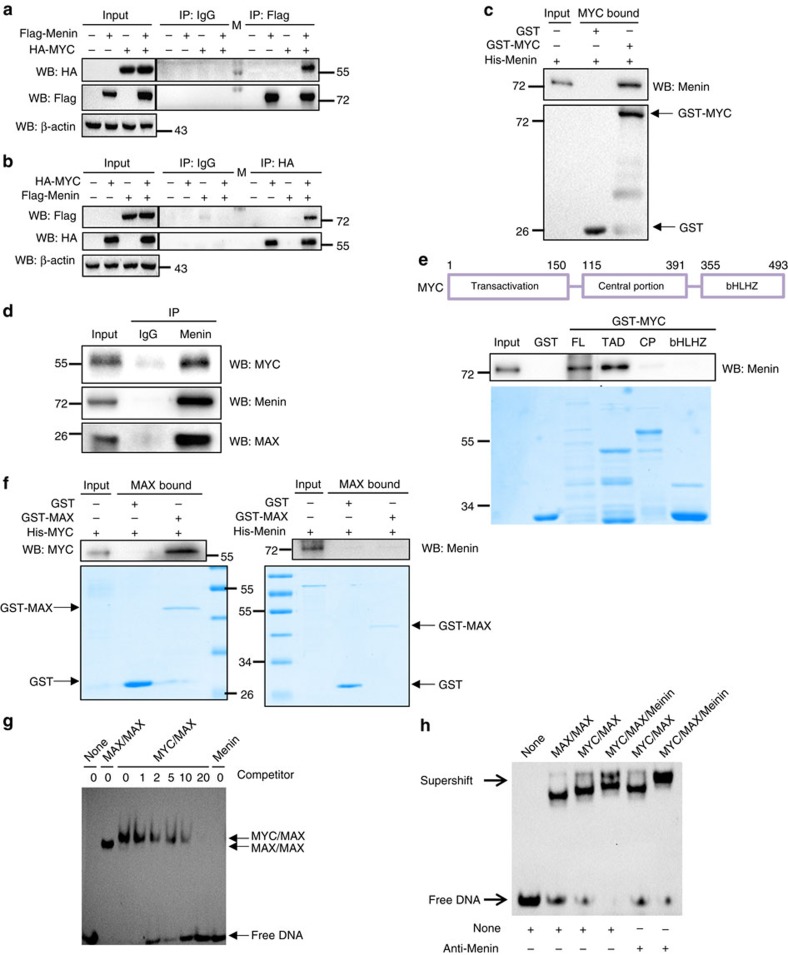
Menin binds to E-box through interacting with MYC. (**a**,**b**) HEK293T cells were transfected with Flag-Menin or HA-MYC or both vectors. IP was performed with anti-Flag (**a**) or anti-HA (**b**), followed by immunoblot analysis. M: protein marker. (**c**) GST pull-down assay was performed with GST or GST fused MYC protein and His-Menin protein. (**d**) Endogenous MYC and Menin interaction. IP of endogenous Menin, MYC and MAX proteins from 293T cells lysed in high-salt buffer. IP was performed with anti-IgG or anti-Mein antibody. (**e**) GST or GST fused MYC truncated proteins TAD, CP and bHLHZ were purified (on the bottom) and used for GST pull-down assay with His-Menin. FL, full length. (**f**) GST pull-down assay was performed with GST or GST fused MAX protein and His-MYC (left panel) or His-Menin (right panel). (**g**) EMSA assays of purified MAX/MAX, MYC/MAX or Menin binding to biotinylated canonical CACGTG E-box sequences following competition with decreasing amounts (20-, 10-, 5-, 2- and 1-fold excess) of unlabelled competitor sequences. The higher bands reflect the amounts of labelled E-box bound by MYC/MAX or MAX/MAX and the lower band reflects the amount of unbound E-box DNA. Lane 1: no proteins. Lane 2: MAX/MAX with no competitor DNA. Lane 3: MYC/MAX with no competitor DNA. Lanes 4–8: the effect of adding indicated amounts of competitor DNA fragments containing the canonical E-box. Lane 9: Menin with no competitor DNA. Bottom: free biotinylated DNA. (**h**) EMSA assay analysing the binding of MAX/MAX, MYC/MAX or MYC/MAX/Menin to biotinylated canonical CACGTG E-box sequences in the presence or absence of anti-Menin antibody.

Although Menin is regarded as a critical factor in regulating H3K4me3 modification, previous studies also reported that Menin has H3K4me3-independent functions^33^,^39^,^40^. Our results indicated that H3K4me3 was not involved in Menin-mediated upregulation of MYC target genes. Given the fact that Menin regulated a large number of MYC target genes and that Menin did not directly regulate the expression of MYC itself (Supplementary Fig. 1i,j), we hypothesized that Menin might directly participate in the MYC-mediated transcription process in a way that was independent of H3K4me3. To address our hypothesis, we first performed co-immunoprecipitation experiments in HEK293T cells co-transfected with HA-MYC and Flag-Menin and found that Menin interacted with MYC (Fig. 2a,b). In addition, GST pull-down using recombinant GST-MYC and His-tagged Menin protein revealed the interaction between Menin and MYC (Fig. 2c), indicating that Menin directly bound to MYC. Our IP experiment also demonstrated the interaction between endogenous Menin and MYC (Fig. 2d). Next, we went on to determine which domian(s) of MYC that interacted with Menin. MYC protein contains several major highly conserved functional domains, including transcactivation domain (TAD), central portion domian (CP) and the basic helix–loop–helix leucine zipper domain (bHLHZ)^34^. Using truncated MYC vectors expressing different functional domains of MYC, our results showed that Menin interacted with the TAD domain (Fig. 2e), indicating that Menin may upregulate MYC target genes by binding to the TAD domain of MYC and enhancing MYC transcriptional activity.

As a basic helix–loop–helix transcription factor, MYC forms a heterodimer with MAX and binds to E-box sequences at the promoter regions of its target genes^41^. Since Menin bound to TAD domain of MYC and Menin elevated the expression of MYC target genes, we next studied whether Menin interacted with MAX or even E-box sequences directly to influence MYC-mediated gene transcription. GST pull-down experiments using recombinant GST-MAX and His-MYC or His-Menin proteins revealed that, unlike MYC dimerizing with MAX (Fig. 2f, left panel), Menin showed no direct association with MAX (Fig. 2f, right panel). Furthermore, electrophoretic mobility shift assay (EMSA) showed no direct binding of Menin to the DNA sequence containing the canonical E-box (CACGTG) (Fig. 2g). In the supershift assay, as expected, E-box was found directly interacting with MYC/MAX heterodimer as well as MAX/MAX homodimer (Fig. 2h). Interestingly, Menin was found to bind to the E-box sequence only in the presence of both MYC and MAX (Fig. 2h), suggesting that Menin bound to canonical E-box sequence in MYC target genes in a way that was mediated by MYC and MAX. Collectively, our results demonstrated that Menin promoted MYC target gene expression by binding to E-box sequence via MYC.

### Menin and MYC colocalize on chromatin

**Figure 3:**
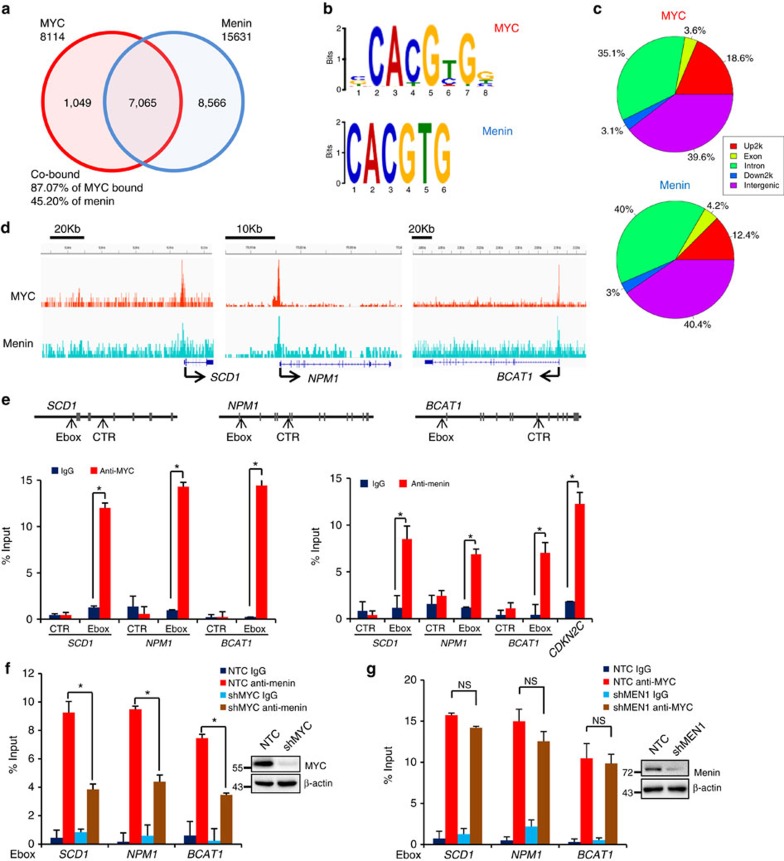
Menin and MYC colocalize on chromatin. (**a**) Venn diagram of the ChIP-seq data showing the overlap of genes bound by MYC and Menin. (**b**) Motif enrichment analysis of the sequences at gene promoters from MYC and Menin ChIP-seq data. Consensus E-box sequences (CACGTG) were significantly enriched from both MYC (*P*-value <1.5 × 10^−121^) and Menin (*P*-value <3.4 × 10^−18^) ChIP-seq peaks. (**c**) Pie graph illustrating genomic locations of MYC and Menin peaks. (**d**) IGV graph showing gene traces of representative genes. (**e**) ChIP experiments were performed in HT1080 cells using IgG or anti-MYC or anti-Menin antibody. The occupancy of potential E-Boxes in *SCD1, NPM1, BCAT1* genes by MYC or Menin was determined by qRT–PCR. Control (CTR) primer sets were also included. *CDKN2C* serves as a positive control for Menin target gene. Data were presented as mean (±s.d.) of three independent experiments. *P<0.05 as compared to corresponding IgG group. (**f**,**g**) ChIP experiments were performed in HT1080 cells expressing sh*MYC* using IgG or anti-Menin antibody (**f**) or in HT1080 cells expressing sh*MEN1* using IgG or anti-MYC antibody (**g**). The occupancy of E-Boxes was determined by qRT–PCR. Data were presented as mean (±s.d.) of three independent experiments. *P<0.05 as compared to between indicated groups.

Since our results indicated that Menin bound to E-box via MYC, we performed ChIP-seq assays for MYC- and Menin-binding sequences to address whether Menin and MYC share some common binding sites on chromatin. First, we analysed our MYC ChIP-seq data set from HT1080 and compared it with a published MYC ChIP-seq data set from P493-6 cells^13^. The results showed that more than 60% of bound genes were overlapped between these two data sets (Supplementary Fig. 3a), indicating that our ChIP-seq data from HT1080 were credible. Interestingly, analysis of the binding sites of MYC and Menin revealed that 87.07% (7065/8114) of MYC-bound gene sequences were also associated with Menin (Fig. 3a). Motif enrichment analysis of sequences at promoters revealed significant enrichment of consensus E-box sequences (CACGTG) in both MYC- and Menin-binding sites (Fig. 3b). Further analysis of the distribution of MYC- and Menin-binding sites on chromatin showed highly similar patterns, confirming that MYC and Menin indeed colocalized on chromatin (Fig. 3c). However, the distribution pattern of H3K4me3-binding sites on chromatin was obviously different from that of MYC and Menin (Supplementary Fig. 3b).

Further analysis of these high throughput sequencing data demonstrated that MYC and Menin co-bound genes were involved in purine metabolism (*PPAT*, *PAICS*), cellular biosynthesic (*NPM1*), cell metabolism (*SCD1*, *BCAT1*) (Supplementary Fig. 3c) and so on. Gene trace analysis also revealed that both MYC and Menin bound to *SCD1*, *NPM1*, *BCAT1*, *PPAT* and *PAICS* genes (Fig. 3d and Supplementary Fig. 3d). To demonstrate whether Menin interacted with MYC at E-box sequences of those genes, we performed ChIP analysis in HT1080 cells using anti-MYC or anti-Menin antibody. The ChIP results showed that both MYC and Menin bound to E-boxes of *SCD1*, *NPM1* and *BCAT1*, confirming that MYC and Menin colocalized on chromatin (Fig. 3e). *CDKN2C*, a known Menin target gene was included as a positive control^42^,^43^ (Fig. 3e, right panel). Interestingly, when MYC expression was blocked in HT1080 cells by shRNAs, the occupancy of those three E-box motifs by Menin was significantly attenuated (Fig. 3f), indicating that MYC was required for Menin to occupy the E-box sequences. Of note, Menin knockdown in HT1080 cells did not affect the binding of MYC to E-box sequences (Fig. 3g).

### Menin promotes the interaction between MYC and P-TEFb

**Figure 4:**
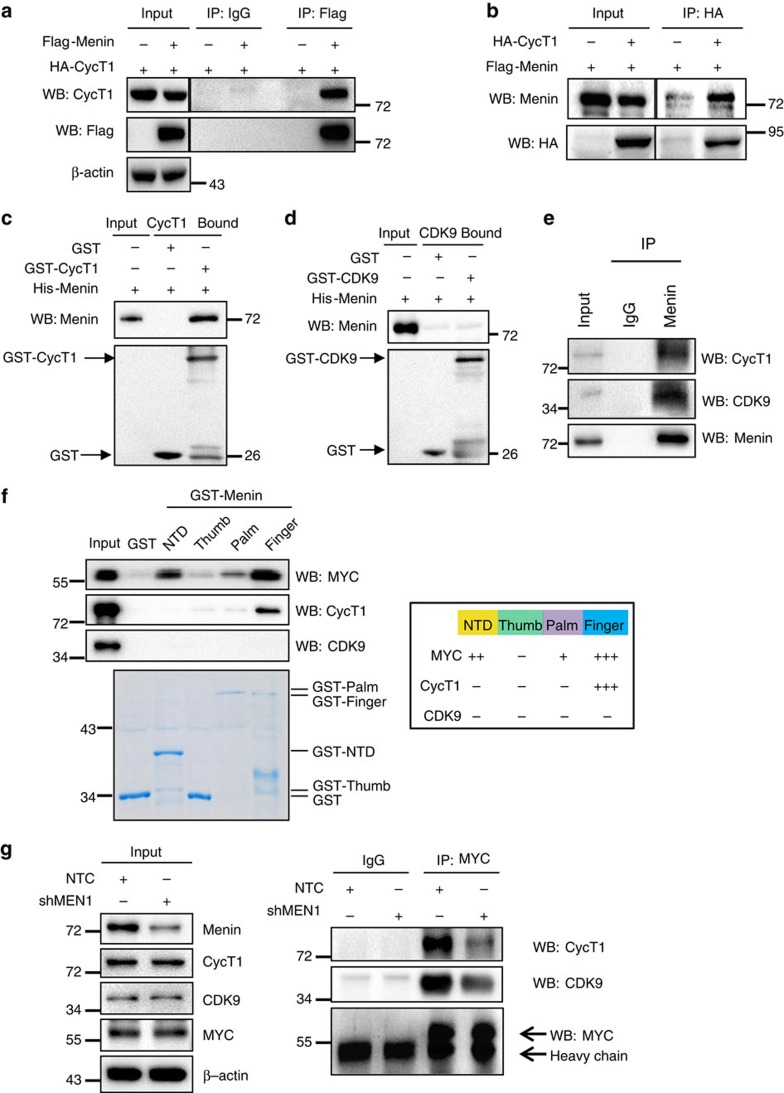
Menin promotes the interaction between MYC and P-TEFb. (**a**,**b**) HEK293T cells were transfected with Flag-Menin or HA-CycT1 or both vectors. IP was performed with anti-Flag (**a**) or anti-HA antibody (**b**), followed by immunoblot analysis. (**c**,**d**) GST pull-down assay was performed with GST or GST fused CycT1 protein and His-Menin (**c**) or GST fused CDK9 protein and His-Menin (**d**). (**e**) Interaction between endogenous Menin and P-TEFb. IP of endogenous Menin, CycT1 and CDK9 proteins from 293T cells lysed in high-salt buffer. IP was performed with anti-IgG or anti-Menin antibody. (**f**) GST or GST fused NTD, Thumb, Palm and Finger domains of Menin proteins were purified (on the bottom of the left panel), and used for GST pull-down assay with His-MYC, His-CycT1 or His-CDK9, respectively. Right panel: summary of the interactions of Menin with MYC or CycT1 or CDK9. (**g**) Interaction between endogenous MYC and P-TEFb. IP of endogenous MYC, Cyclin T1 and CDK9 proteins from 293T that transfected with NTC or sh*MEN1* for 48 h. IP was performed with anti-IgG or anti-MYC antibody.

Our data showed that Menin bound to E-box sequences through TAD domain of MYC (Figs 2e and 3e,f); however, Menin suppression did not affect the occupancy of MYC to the E-box sequences (Fig. 3g). Thus, we next asked how Menin regulated MYC transactivity. Previous reports have shown that MYC occupancy at the promoters of target genes could recruit P-TEFb and thereby enhance elongation by RNA Pol II in tumour cells^44^,^45^,^46^,^47^. Recently, it has been found that, by binding to P-TEFb, MYC also regulates transcriptional pause release^11^ and amplifies transcription by activating RNA Pol II in an H3K4me3-independent manner^10^. Hence, we next investigated whether Menin affected the recruitment of the P-TEFb complex during MYC-regulated transcription. P-TEFb is a heterodimer that consists of Cyclin T1 (CycT1) and cyclin-dependent kinase CDK9. Co-immunoprecipitation experiments in HEK293T cells overexpressing exogenous Menin and CycT1 revealed that Menin interacted with CycT1 (Fig. 4a,b). In addition, recombinant His-Menin protein also bound to purified GST-CycT1 protein (Fig. 4c), indicating that Menin directly interacted with CycT1. Not surprisingly, recombinant His-Menin protein did not directly bind to purified GST-CDK9 protein, the catalytic subunit (Fig. 4d). IP experiment also revealed the interaction between endogenous Menin and P-TEFb (Fig. 4e). Of note, to rule out the possibility that the interaction of Menin and MYC, P-TEFb might depend on co-extracted DNA, we performed the endogenous IPs side-by-side in low-salt (150 mM) and high-salt (300 mM) IP buffers. Our results showed that, similar to the results of the endogenous IPs in low-salt buffers, endogenous Menin also interacted with MYC and P-TEFb in high-salt buffers (Supplementary Fig. 4), indicating that the interaction of Menin and MYC, P-TEFb is independent of co-extracted DNA. Taken together, our data demonstrated that Menin interacted directly with both MYC and P-TEFb complex.

Menin contains a deep pocket and can be divided into four different domains, including NTD (N-terminal domain), Thumb, Palm and Finger domains^48^. To further clarify the interaction among Menin, MYC and P-TEFb complex, we purified GST-fused Menin protein fragments including GST-NTD, GST-Thumb, GST-Palm and GST-Finger domains and performed the pull down experiments with His-MYC, His-CycT1 and His-CDK9, respectively (Fig. 4f). We found that MYC had a strong binding to the Finger domain but weak binding to NTD and Palm domains, while the P-TEFb complex, CycT1, specifically interacted with the Finger domain (Fig. 4f). Consistent with the data in Fig. 4d, no fragments of Menin interacted with CDK9 (Fig. 4f). More interestingly, IP experiment results showed that the interaction between endogenous MYC and P-TEFb complex was significantly inhibited when Menin was knocked down by shRNAs (Fig. 4g), suggesting that Menin was required for the interaction between MYC and P-TEFb.

### Menin enhances MYC activity via P-TEFb

**Figure 5:**
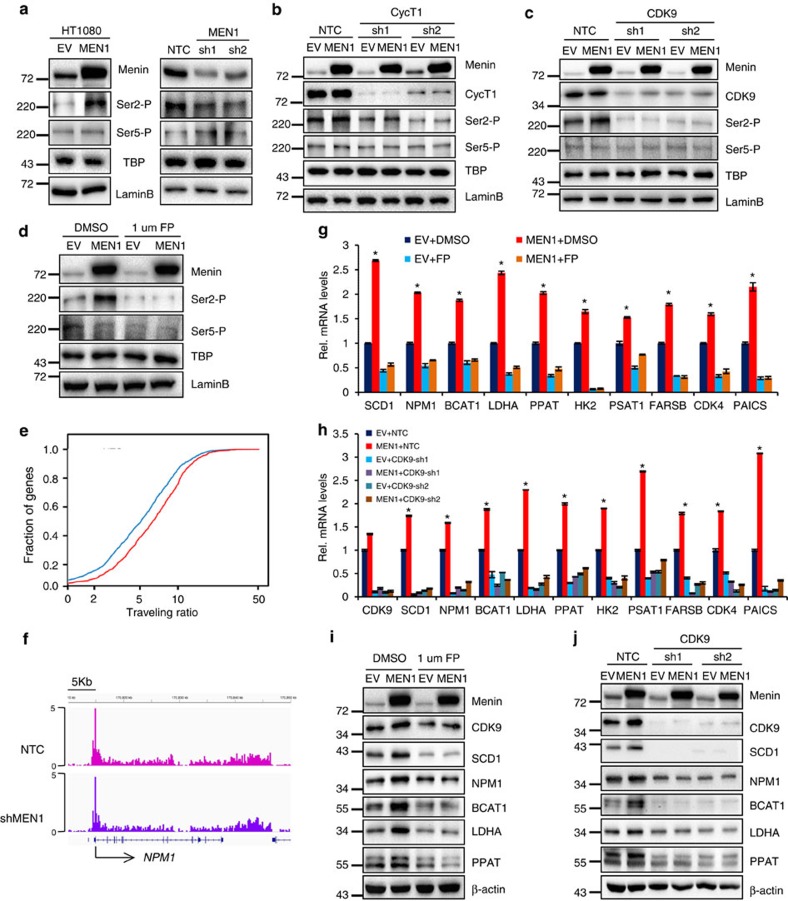
Menin requires P-TEFb to transcriptionally enhance the expression of MYC target genes. (**a**) Western blotting analysis for the modified forms of RNA Pol II using specific antibodies against Pol II Ser2-P CTD or Pol II Ser5-P CTD in HT1080 cells stably expressing exogenous Menin (left panel) or shMEN1 (right panel) from nuclear lysate. TBP and laminB serve as loading controls for RNA Pol II and total nuclear protein, respectively. (**b**–**d**) Western blotting analysis for the modified forms of RNA Pol II in Menin overexpressing HT1080 cells in the presence of sh*CycT1* (**b**), sh*CDK9* (**c**) or CDK9 inhibitor (1 μM of FP) (**d**) from nuclear lysate. TBP and laminB serve as loading controls for RNA Pol II and total nuclear protein, respectively. (**e**) Empirical cumulative distribution plots of RNA Pol II traveling ratio for 1,000 transcribed genes. Genes were randomly selected from RNA Pol II ChIP-seq data. Welch’s two-tailed *t*-test, *P*-value <2.52 × 10^−10^. (**f**) IGV graph showing gene traces of RNA Pol II occupancy at *NPM1* gene. (**g**) qRT–PCR assay for mRNA expression of MYC target genes in Menin overexpressing HT1080 cells in the presence of CDK9 inhibitor (1 μM of FP). Data were presented as mean (±s.d.) of three independent experiments. *P<0.05 as compared to EV+DMSO group. (**h**) qRT–PCR assay for mRNA expression of MYC target genes in Menin overexpressing HT1080 cells that were further infected with virus expressing sh*CDK9*. Data were presented as mean (±s.d.) of three independent experiments. *P<0.05 as compared to EV+NTC group. (**i**,**j**) Western blotting assay for the expression of Menin, MYC target genes in Menin-overexpressing HT1080 cells in the presence of CDK9 inhibitor (1 μM of FP) (**i**) or in Menin-overexpressing HT1080 cells which were further infected with virus expressing sh*CDK9* (**j**). β-Actin serves as a loading control.

We next asked if P-TEFb was required for Menin to enchance MYC-mediated transcription. P-TEFb predominantly phosphorylates the second serine (Ser2) residue of the RNA Pol II C-terminal domain (CTD)^49^, enhancing the elongation progress. Our data showed that phosphorylation at Ser2 (Ser2-P), but not phosphorylation at Ser5 (Ser5-P), of RNA Pol II-CTD was increased by Menin overexpression and decreased by Menin shRNAs (Fig. 5a), suggesting that Menin was involved in RNA Pol II-mediated elongation. Further analysis revealed that when CycT1 or CDK9 were suppressed by shRNAs, the effect of Menin on Ser2-P was eliminated (Fig. 5b,c), suggesting that Menin activated RNA Pol II via P-TEFb. Furthermore, when flavopiridol (FP), a CDK9 kinase inhibitor^50^,^51^, which could efficiently block the Ser2-P level of RNA Pol II-CTD (Fig. 5d and Supplementary Fig. 5a), was used in HT1080 cells, Menin-enchanced Ser2-P level was significantly attenuated (Fig. 5d), further suggesting that Menin activated RNA Pol II via P-TEFb.

We next studied the effect of Menin on RNA Pol II-mediated transcriptional elongation by performing RNA Pol II ChIP-seq data analysis. Our data documented that suppression of Menin by shRNAs resulted in a significant decrease in elongating RNA Pol II levels across the genome as measured by traveling ratio (TR) (*P*–value <2.52 × 10^−10^) (Fig. 5e and Supplementary Fig. 5b). As expected, genes *NPM1* and *BCAT1*, for examples, displayed an obvious decrease in elongating RNA Pol II levels when Menin was knocked down (Fig. 5f and Supplementary Fig. 5c). In addition, we performed ChIP assays to study the recruitments of CDK9 and Ser2-phosphorylated RNA Pol II at MYC target genes in HT1080 cells with *MEN1* knockdown. As a result, when Menin was suppressed in HT1080 cells by shRNAs, the recruitment of CDK9 to MYC target genes was significantly attenuated, so was Ser2P (Supplementary Fig. 5d–f), indicating that Menin is required for the recruitments of both CDK9 and Ser2-phosphorylated RNA Pol II at MYC target genes. These results supported that Menin facilitated the interaction between MYC and P-TEFb to stimulate the RNA Pol II elongation at MYC target genes. Moreover, CDK9 inhibition with FP treatment significantly attenuated Menin-enhanced MYC target gene expression at both mRNA and protein levels (Fig. 5g,h), indicating that CDK9 activity was critical for Menin-enhanced MYC target gene expression. Knocking down CDK9 or CycT1 markedly inhibited MYC target gene expression both at mRNA and protein levels (Supplementary Fig. 6a–d). Consistently, suppression of CDK9 by shRNAs abolished the enhancing effect of Menin on MYC target gene expression both at mRNA and protein levels (Fig. 5i,j), further confirming that P-TEFb was critical for Menin-enhanced MYC target gene expression. Collectively, our data demonstrated that Menin enhances MYC-mediated transcription by stimulating RNA Pol II via P-TEFb.

### Menin promotes MYC-mediated cancer metabolism and growth

**Figure 6:**
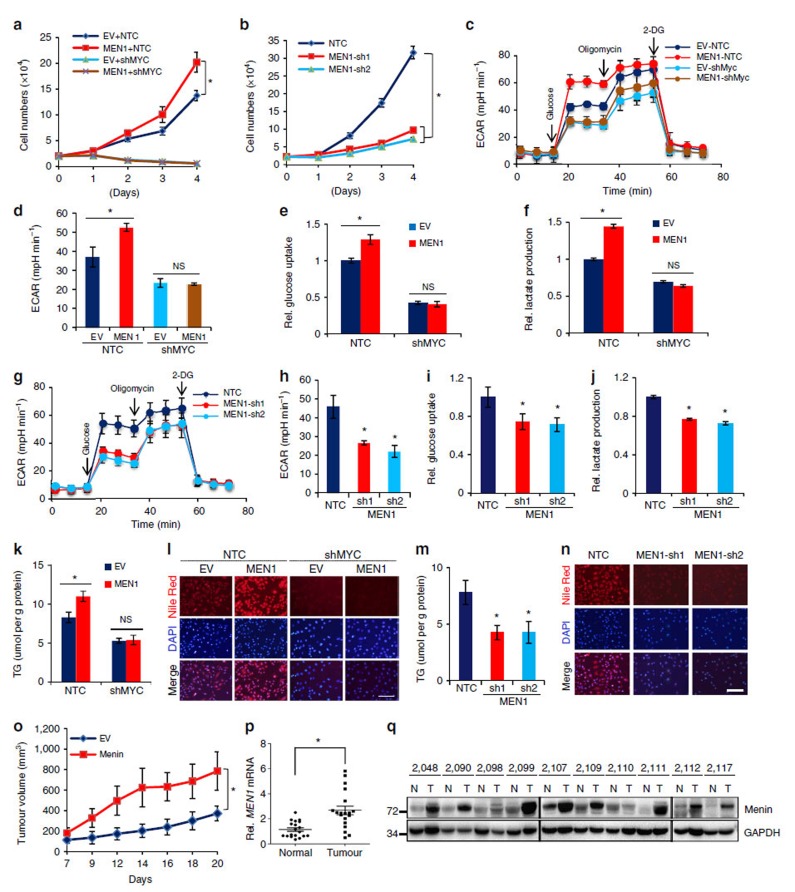
Menin is critical for MYC-mediated cell metabolism and cancer progression. (**a**,**b**) Growth curves were analysed in HT1080 cells overexpressing Menin and further infected with viruses expressing sh*MYC* (**a**) and HT1080 cells expressing NTC or sh*MEN1* (**b**) by trypan blue counting. (**c**,**d**,**g**,**h**) Glycolysis profiling as in **a**,**b**. ECAR rate (a proxy for the rate of glycolysis) was used to assess the glycolysis using a Seahorse 96XF extracellular flux analyzer following sequential addition of glucose (10 mM), oligomycin (1.0 mM) and 2-DG (50 mM) as arrows indicated (**c**,**g**). ECAR were analysed by Seahorse XF Glycolysis Stress Test Report Generator (**d**,**h**). (**e**,**f**,**i**,**j**) Glucose uptake (**e**,**i**) and lactate production (**f**,**j**) were measured as in **a**,**b**. (**k**,**m**) Cellular TG contents were measured by kit as in **a**,**b**. Values were normalized to cellular protein. Scale bars, 100 μm (**l**). (**l**,**n**) Lipid storage were measured by Nile red/DAPI staining as in **a**,**b**. Scale bars, 100 μm. (**o**) HT1080 cells stably expressing EV or Menin were injected subcutaneously into nude mice (*n*=5 for each group). Tumour growth curves were measured starting from 7 days after inoculation. Data were presented as mean (±s.e.m.). (**p**) *MEN1* mRNA were determined by qRT–PCR assay in 19 pairs of clinically matched tumour adjacent non-cancerous liver tissues (normal) and human hepatocellular carcinoma (HCC) tissues (tumour). mRNA levels were normalized to 18S rRNA. (**q**) Menin protein levels were determined by western blot using the paired tumour adjacent non-cancerous liver tissues (normal) and human HCC tissues (tumour). GAPDH serves as a loading control. Result is representative of three independent experiments. Error bars correspond to s.d. except for (**o**). *P<0.05 as compared between indicated groups using Student’s *t*-test. NS, not significant.

Since our data established that Menin affected the expression of a large number of MYC target genes, such as *CDK4* for cell cycle, *LDHA* and *HK2* for glycolysis and *SCD1* for lipid metabolism, we next performed experiments to determine the effects of Menin on MYC-mediated cell proliferation, glycolysis, lipid metabolism and tumour growth. MYC is highly expressed in most cancer cells, including HT1080 cells. Overexpression of Menin promoted cell proliferation in HT1080 cells; however, when MYC was suppressed by shRNA, the effect of Menin on cell proliferation was eliminated (Fig. 6a), suggesting that Menin enhanced cell proliferation via MYC. Consistently, Menin shRNAs significantly impaired cell proliferation (Fig. 6b). Our data demonstrated that Menin promoted cell proliferation via MYC.

MYC is involved in regulating many metabolic enzymes, such as LDHA and HK2 for glycolysis, in cancer cells. Our results showed that LDHA and HK2 were also regulated by Menin (Fig. 1c). In HT1080 cells which have high basal level of MYC, overexpression of Menin increased cell glycolysis as measured by the extracellular acidification rate (ECAR) (Fig. 6c,d) and glucose uptake and lactate production (Fig. 6e,f), indicating that Menin enhanced glycolytic metabolism in cancer cells. However, when MYC was suppressed by shRNA, the effect of Menin on glycolysis was abolished (Figs 5f and 6c), suggesting that Menin-enhanced glycolysis was mediated by MYC. In addition, Menin knockdown significantly decreased glycolysis in HT1080 cells (Fig. 6g–j).

SCD1, an MYC target gene regulated also by Menin (Fig. 1c), is a crucial enzyme in lipid synthesis and fat storage^52^,^53^. As previously reported^54^, our data also showed that inhibition of SCD1 by shRNAs impaired cell proliferation in P493-6 cells and in HT1080 cells (Supplementary Fig. 7a,b) and suppressed lipid synthesis with decreased cellular triglyceride (TG) content and reduced Nile red staining for lipid accumulation in HT1080 cells (Supplementary Fig. 7c,d). Consistently, we observed that MYC knockdown led to decreased cellular TG content and reduced Nile red staining (Supplementary Fig. 7e,f), confirming that MYC regulated lipid metabolism. In addition, forced expression of Menin increased cellular TG content and enhanced Nile red staining in HT1080 cells, which was abolished by MYC shRNA (Fig. 6k,l), demonstrating that Menin-regulated lipid metabolism was mediated by MYC. Furthermore, Menin knockdown decreased TG content and reduced Nile red staining in HT1080 cells (Fig. 6m,n). Thus, our data revealed that Menin regulated cancer cell metabolism via MYC.

Since our *in vitro* results showed that Menin affected the metabolism and proliferation of HT1080 cells in an MYC-dependent manner, we next studied if Menin regulated tumour growth *in vivo*. Mouse xenograft study using HT1080 cells stably overexpressing Menin or empty vector (EV) control revealed that forced expression of Menin markedly accelerated tumour growth *in vivo* (Fig. 6o and Supplementary Fig. 7g,h). Western blot analysis using protein lysate from xenograft tumours confirmed the overexpression of Menin (Supplementary Fig. 7i). In addition, consistent with the *in vitro* data, TG content in tumour tissues was increased in the Menin-overexpressing group (Supplementary Fig. 7j). Thus, our results from both *in vitro* and *in vivo* experiments demonstrated that Menin promoted cancer progression. To further address whether Menin was related with human malignancies, we employed the clinical human HCC samples and found that Menin was highly expressed in HCC compared to adjacent normal tissues both at mRNA and protein levels (Fig. 6p,q and Supplementary Fig. 7k), suggesting a strong correlation of Menin expression with clinical HCC. These results were consistent with previous reported roles for Menin as an oncoprotein^17^,^18^. Overall, our data demonstrated that Menin enhanced the tanscriptional acitivity of MYC to promote cancer progression.

## Discussion

**Figure 7:**
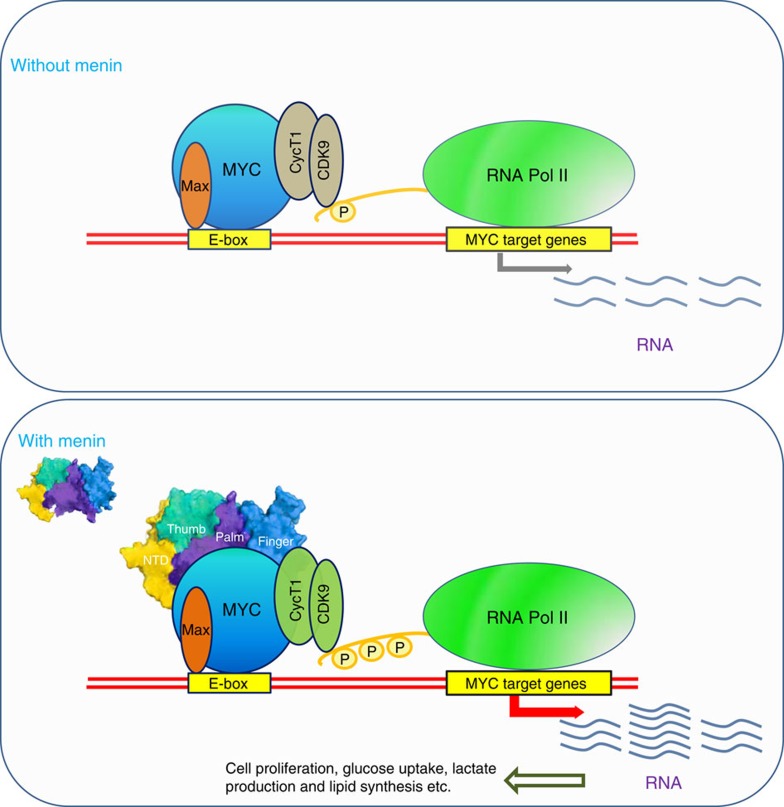
Working model: Menin enhances MYC-mediated transcription. It has been reported that MYC binds to P-TEFb and acts as an amplifier for gene expression. Here, we depict that Menin, an enigmatic protein that has been documented to both promote and suppress tumorigenesis, enhances MYC-mediated transcription. Without Menin, the expression of MYC target genes is feeble (top). In the presence of high Menin, it recruits P-TEFb to MYC, facilitating RNA Pol II phosphorylation, leading to enhanced MYC-mediated transcription and promoting MYC-mediated cell proliferation, cancer metabolism and cancer progression (bottom).

There has been tremendous interest recently in the way MYC regulates gene transcription and tumorigenesis^5^,^10^,^12^,^13^,^14^,^34^,^55^,^56^,^57^,^58^,^59^,^60^. Traditional paradigm has long established MYC as a multifaceted transcriptional factor to regulate gene expression by binding to the promoter regions of specific target genes^5^,^61^. This paradigm has been challenged recently by reports proposing that, instead of regulating a specific set of genes, MYC amplifies the extant active gene expression in mammalian cells^10^,^12^. More interestingly, two recent studies provided new evidence documenting that MYC selectively modulates transcription of genes that shape the cellular growth and tumorigenesis^13^,^14^. Nevertheless, those seemingly different results do agree on several aspects: firstly, MYC binds to the promoter and enhancer regions of target genes; secondly, MYC-mediated transcription involves other transcriptional factors or cofactors that may determine the outcome of the transcriptional regulation. Obviously, one gap in our understanding of MYC-regulated transcription mechanism currently lies in the identification of other transcription factors/cofactors or new mechanisms that may be involved in this process. In this regard, we uncovered here that Menin is critical for MYC-mediated transcriptional regulation (Fig. 7). We showed that Menin interacts with the TAD domain of MYC directly and binds to E-boxes to enhance transcription of MYC target genes. We further identified that Menin enhances transcription of MYC target genes in a way dependent on P-TEFb, which has been revealed recently as a key factor to facilitate transcription amplification by MYC. Moreover, we demonstrated that, by transcriptionally promoting the expression of MYC target genes in cancer cells, Menin stimulates cell proliferation, cellular metabolism and cancer progression. Taken together, our results reveal a previously unappreciated mechanism by which Menin functions as a regulatory factor that is critical for MYC-mediated transcription and cancer development.

The establishment of a link between Menin and MYC in tumorigenesis is very interesting. Previously, Bres *et al*.^33^ observed that, during HIV infection, Menin could interact with MYC, but its impact for oncogenic activity has never been experimentally explored. Here, surprisingly, we observed that, in cancer cells, Menin enhanced the expression of a large number of MYC target genes by interacting with MYC and P-TEFb. This observation establishes Menin as an oncogenic factor to promote tumour growth at least when MYC expression is deregulated. On the one hand, as accumulating evidence suggests differential roles for Menin in cancer cells, we report here a novel mechanism underlying Menin’s oncogenic activity; on the other hand, our results bring in Menin to the complicated MYC-regulatory models to demonstrate that Menin is a critical cofactor for MYC-mediated transcription. Previously, Menin was implicated in tumorigenesis of liver and haematopoietic system by serving as an essential oncogenic cofactor for MLL1 and MLL2, which are HMTs that mediate H3K4me3 (refs 17, 18, 19, 20). However, recent evidence suggest that these MLL cofactors might have broader effects than serving as HMTs. Ullius *et al*.^60^ reported recently that ASH2L, an MLL subunit, interacts directly with the bHLHZ domain of MYC and promotes gene transcription by modifying H2K27 acetylation. More recently, Thomas *et al*.^59^ reported that interation with WDR5 could promote target gene recognition and tumorigenesis by MYC interacting with MB IIIb domain of MYC box motif in CP domain. They also showed that the genomewide profiles of MYC and WDR5 are noticeably different from that for H3K4 trimethylation^59^, which is consistent with our finding that H3K4 trimethylation is not involved in the enhanced transcription of MYC target genes by Menin. Here we provide ample evidence to show that Menin interacted with the TAD domain of MYC to promote MYC-mediated transcriptional elongation. Interestingly, we showed that this unexpected function of Menin was not dependent on its role as a component of the MLL HMT complex as knockdown of two other MLL components ASH2L and RBBP5 did not recapitulate similar effect on MYC-mediated gene transcription, suggesting that Menin is more widely involved in tumorigenesis that warrants more in-depth investigation.

Past several decades have seen the rapid expansion in understanding the functions of MYC in cell growth and cancer progression^1^. While some have suggested that there seems no pattern for MYC-regulated genes^10^,^12^, recent evidence indicates that the models for MYC regulation could be context dependent: MYC exerts different roles under normal development and oncogenic conditions^13^,^62^. Interestingly, the same context-dependent regulation is likely to be true with the activity of Menin, which serves both as a tumour suppressor and oncoprotein, pending on different circumstances. The question then is how, and under what context, this regulation is determined. While the current study cannot answer this big question in full, we did provide evidence to demonstrate that Menin and MYC are mutually dependent and cooperatively promote cancer progression. As MYC is known to be deregulated in 30–50% of human malignancies with poor prognosis, our novel discovery may have significant implication for cancer therapy: targeting both Menin and MYC pathways might potentially work for human malignancies with aberrant expression of both MYC and Menin.

## Methods

### Cell culture and reagents

HT1080 and HepG2 were purchased from the Type Culture Collection of Chinese Academy of Sciences, Shanghai, China. HT1080, HepG2 and HEK293T cells (from ATCC) were cultured in Dulbecco’s modified Eagle’s medium DMEM (Gibco). P493-6 B cells (a gift from Dr Chi V. Dang) were cultured in RPMI-1640 medium (Gibco). All cell lines were tested for mycoplasma contamination and no cell lines were contaminated. Both DMEM and RPMI-1640 were supplemented with 10% FBS (Gibco) and 1% penicillin–streptomycin (Gibco). Cells were maintained in an incubator with 5% CO_2_ at 37 °C. To repress MYC expression in P493-6 B cells, 0.1 μg ml^−1^ tetracycline (Sigma) was added into the culture medium. To repress CDK9 activity, FP (Sigma) was used.

### Plasmids and established stable cells

pBabe-GFP empty vector or pBabe-GFP-MYC vector^63^, pMX-T7-2xFlag empty vector or pMX-T7-2xFlag-Menin vector (gift from Dr Xianxin Hua in University of Pennsylvania)^64^ was co-transfected with plasmids encoding gag/pol and VSVG into HEK293T packaging cells using lipofectamine 2000 (Invitrogen). HT1080 cells were infected with produced retrovirus in the presence of polybrane and selected with 0.5 mg ml^−1^ G418 or 0.5 μg ml^−1^ puromycin to establish stale cells. Lentiviral shRNAs targeting human *MYC*, *MEN1*, *ASH2L*, *RBBP5*, *CDK9*, *Cyclin T1* and *SCD1* were purchased from Sigma. shRNA targeting sequences are listed in Supplementary Table 1. Viruses expressing shRNAs were produced in HEK293T cells and infected HT1080 or HepG2 cells in the presence of polybrane.

### RNA extraction and quantitative real-time PCR

Total RNA was isolated using Trizol reagent (Life technologies) and followed by DNase (Ambion) treatment. One microgram of RNA was used to synthesize cDNA by the iScript cDNA Synthesis Kit (Bio-Rad). Sequences of used primer are showed in Supplementary Table 2. Quantitative real-time PCR was performedusing iQ SYBR Green Supermix and the iCycler Real-time PCR Detection System (Bio-Rad). mRNA levels were compared to 18S rRNA and the fold change of target mRNA expression was calculated based on threshold cycle (Ct), where ΔCt=Ct_target_−Ct_18S_ and Δ(ΔCt)=ΔCt_Control_−ΔCt_Indicated condition_.

### Western blot

Cells were harvested and proteins were extracted using RIPA buffer (50 mM Tris-Cl, pH 8.0, 150 mM NaCl, 5 mM EDTA, 0.1% SDS, 1% NP-40) supplemented with protease inhibitor cocktail. Proteins were quantified with Bradford assay kit (Sangon Bio). Equal amounts of proteins were fractionated by 5–12% SDS–PAGE. The following primary antibodies were used: Menin (1:2,000; A300-105A) (Bethyl Laboratories, Montgomery, USA); c-Myc (9E10) (1:1,000; MA1-980) (Zymed, San Francisco, USA); SCD (1:2,000; ab19862), H3 (1:2,000; ab1791), BCAT1 (1:500; ab195663) (Abcam, Cambridge, USA); H3K4me3 (1:2,000, #9751), c-Myc (1:2,000, 9402s) (Cell Signaling Technology, Beverly, MA, USA); CyclinT1 (1:1,000; 20992-1-AP), CDK9(1:1,000; 11705-1-AP) ASH2L (1:1,000; 12331-1-AP) TBP(1:2,000; 22006-1-AP), RBBP5 (1:1,000; 13181-1-AP), PPAT (1:1,000; 15401-1-AP), NPM1 (1:1,000; 60096-1-Ig), LDHA (1:1,000; 21799-1-AP), GST (1:5,000; 10000-0-AP), β-actin (1:5,000; 66009-1-Ig), GAPDH (1:2,000; 18142-1-AP) (Proteintech, Rosemont, IL, USA); Serine 2 phospho of CTD (1:1,000; MMS-129R), serine 5 phospho of CTD (1:1,000; MMS-134R) (Covance, Dedham, MA, USA); MAX (1:1,000; sc-764), laminB (1:2,000; sc-6216) (Santa Cruz Biotechnology, Santa Cruz, USA); Flag-M2 (1:5,000; F1804) and HA (1:5,000; H9658) (Sigma-Aldrich, St Louis, MO, USA). HRP-conjugated anti-rabbit and anti-mouse (1:10,000; Bio-Rad) secondary antibodies were used. Signalling was detected by Western ECL Substrate (Bio-Rad).

Uncropped images of immunoblots presented in this paper are provided in Supplementary Fig. 8.

### Immunoprecipitation

For co-immunoprecipitation, cells were lysed with low-salt IP buffer (0.5% NP-40, 20 mM HEPES (pH 7.5), 150 mM NaCl, 2 mM EDTA, 1.5 mM MaCl_2_) supplemented with protease inhibitor cocktail for 30–40 min on ice, and centrifuged at 16,000 *g* for 10 min at 4 °C. The supernatants were incubated with indicated antibody overnight at 4 °C followed by incubation with protein A/G conjugated beads for 2 h. Beads were then washed with IP buffer and boiled in SDS-loading buffer and analysed by western blot.

For endogenous immunoprecipitation, 6 × 10^7^ cells were lysed with low-salt buffer or high-salt IP buffer (0.5% NP-40, 20 mM HEPES (pH 7.5), 300 mM NaCl, 2 mM EDTA, 1.5 mM MaCl_2_) and followed by IP as described above.

### Fusion protein pull-down experiment

The cDNAs coding *MYC*, *MEN1*, *CDK9*, *Cyclin T1* or *MAX* were cloned into pET-22b (+) vector (Novagen) and cDNAs coding *MYC*, *CDK9*, *Cyclin T1*, *Menin-NTD*, *Menin-Thumb*, *Menin-Palm*, *Menin-Finger, MYC-TAD, MYC-CP* and *MYC-bHLHZ* were cloned into pGEX-4T-1 (GE) vector by ClonExpressTM II One Step Cloning Kit (Vazyme). Proteins were produced in *Escherichia coli* (DE3). Purified GST fused proteins and His-tag proteins were used for pull-down experiments in pull-down buffer (PDB) (150 mM NaCl, 50 mM Tris (pH 7.5), 0.1% NP-40, 5 mM DTT). After incubation, the beads were pelleted and washed with PDB buffer followed by elution of proteins and analysis by western blot.

### RNA-seq analysis

Total RNA was extracted using Trizol Reagent (Life technologies) following the manufacturer’s instructions and checked for an RIN number to inspect RNA integrity by an Agilent Bioanalyzer 2100. A total amount of 3 μg RNA per sample was used as input material for the RNA sample preparations. Sequencing libraries were generated using NEBNext Ultra RNA Library Prep Kit for Illumina (NEB, USA) following the manufacturer’s recommendations and index codes were added to attribute sequences to each samples. RNA was sequenced by Novogene (Beijing, China) using the Illumina Hiseq4000 platform. Reads were alligned to the human genome hg19 with BOWTIE v2.2.3. Gene expression analysis was performed using the DEGSeq R package (1.26.0). Unsupervised clustering was performed using cluster and tree view and visualized using heat maps. Enrichment pathway analysis of genes was compiled from BIOCARTA and Kyoto Encyclopedia of Genes and Genomes (KEGG) pathway databases by DAVID Bioinformatics Resources. Original data are available in the NCBI Gene Expression Omnibus (GEO) (accession number GSE86504).

### Chromatin immunoprecipitation assay

The ChIP assay was performed with an EZ-ChIP kit (Millipuro) following the manufacturer’s instruction. Briefly, cells were fixed with 1% formaldehyde and quenched in 0.125 M glycine. Cells were sonicated by Bioruptor Sonication System UCD-300. DNA was immunoprecipitated by either control IgG or MYC (Cell Signaling Technology, 9402S), CDK9 (Santa cruz, sc-8338x), Serine 2 of phosphor of CTD (Covance, MMS-1129R) or Menin (Bethyl Lab, A300-105A) primary antibody, followed by quantitative real-time PCR analysis (Bio-rad). The oligos used for this analysis are listed in Supplementary Table 2.

### ChIP-seq and data analysis

The ChIP was performed with an EZ-ChIP kit (Millipuro) following the manufacturer’s instruction as described above. In this experiment, the DNA fragments were sonicated between 100 to 500 bp. For H3K4me3 ChIP, 2 × 10^7^ cells were used. For MYC, Menin, RNA Pol II, 8 × 10^7^ cells were used. Antibodies were used as follows: H3K4me3 (Millipore, 07-473), MYC (Cell signaling Technology, 9402S), Menin (Bethyl lab, A300-105A), RNA Pol II (N-20) (Santa Cruz Biotechnology, sc-899).

ChIP-seq was performed at BGI (Shenzhen, China) and sequencing was performed on the Illumina Hiseq2500 platform. Sequenced reads were mapped to the UCSC human genome hg19 using software Bowtie2 (version 2.2.6). Aligned reads were used for subsequent generation of binding profiles, peak callings, motif analyses and TR analyses. We used the software tool MACS (version 1.4.2) for peak callings from the aligned reads, using the default settings: the band width=300, *P*-value=1e-5, shiftsize=100, fold enrichment step=20. We used annotatePeaks.pl, which contains in HOMER (Hypergeometric Optimization of Motif EnRichment) (version 3.12) to annotate the peaks with the UCSC human genome hg19. Peaks were classified based on the location (UCSC annotation data) and showed in the following genome regions: intergenic, introns, downstream, upstream and exons. The gene traces were visualized using the Integrative Genomics Viewer. MYC- and Menin-binding motif was analysed by MEME. The MYC or Menin enriched regions with ±2 kb of transcribed genes TSS were ranked by MACS and the top 500 enriched regions were used as input for MEME. The default settings in MEME-ChIP were described as follows: the minimum motif window width=6, maximum motif window width=30, minimum sites to accept a motif=2 and maximum motif sites=600. For Fig. 5e and Supplementary Fig. 5b, RNA Pol II TR was determined using RNA Pol II ChIP-seq data as described^10^. We defined the initiating region from −300 to +300 bp relative to the TSS and the elongating region from +300 bp from the TSS to +3,000 bp after the gene end. Original data are available in the NCBI GEO (accession number GSE86504).

### Electrophoretic mobility shift assay

EMSA was performed using recombinant His-MYC, His-MAX and His-Menin proteins following the protocol previously reported^65^. Briefly, purified proteins were added in the EMSA buffer (50 mM HEPES, 150 mM NaCl, 0.2% BSA (w/v), 0.02% Tween 20, 0.5 mM MgCl_2_ and 100 μM DTT) to form indicated complexs. One microlitre of Menin antibody was added to do supershift assay. Biotinylated DNA containing the canonical E-box sequence (CACGTG) was added into those complexes and incubated for 60 min at room temperature. Samples were fractionated on 5% gel and transferred to Nylon membrane (Millipuro). DNA was UV crosslinked to membrane and biotinylated DNA was detected using Pierce Chemiluminescent Nucleic Acid Detection Module (Thermo). The same E-box sequecnces (GGAAGCAGACCACGTGGTCTGCTTCC) were used for biotinlyated DNA and competitor DNA.

### Extracellular acidification rate

Measurements of ECAR in HT1080 cells were performed using the Seahorse XF96^e^ analyzer (Seahorse Bioscience, North Billerica, MA, USA) according to the manufacter’s instruction. Briefly, 1.0 × 10^4^ cells were seeded per well overnight in a 96-well XF cell culture microplate in growth medium. ECAR was measured with an XF96 analyzer in XF base medium containing 4 mM glutamine (pH 7.35) following sequential additions of glucose (10 μM), oligomycin (1 μM) and 2-DG (50 mM). Data were analysed by a Seahorse XF Glycolysis Stress Test Report Generator.

### Glucose uptake and lactate production

The intracellular glucose was measured in the cell lysates by the Glucose Assay Kit (BioVision) and the extracellular lactate was measured in the medium by the Lactate Assay Kit (BioVision), following the manufacturer’s instruction. All the values were normalized to protein concentration.

### Lipid determination

Lipid determination was performed as follows. To visualize lipid droplets, cultured cells were fixed in 4% paraformaldehyde solution on the 12-well plates, stained with 0.05 μg ml^−1^ Nile red (Sigma) for 10 min, washed with PBS twice and then stained with DAPI. The images were visualized by immunofluorescence microscopy. Collected cells were lysed in RIPA buffer with 1% NP-40 for 30 min, and cell lysates were used to measure triglycerideby using a Biochemical Triglyceride Determination Kit (NJJC Bio). The values were normalized to cellular protein.

### Animal studies

All animal studies were conducted with approval from the Animal Research Ethics Committee of the University of Science and Technology of China. For xenograft experiments, HT1080 cells stably expressing empty vector or Menin were injected subcutaneously into 5-week-old male BALb/c nude mice (*n*=5 for each group) (SJA Laboratory Animal Company, China). Seven days after injection, tumour volumes were measured every 2 or 3 days with a caliper and calculated using the equation: volume=width × depth × length × 0.52.

### Clinical human HCC specimens

Human tissue specimens, including 19 pairs of freshly snap-frozen HCC tissues and paired normal adjacent tissues, were histopathologically diagnosed at Yat-sen University Cancer Center (Guangzhou, China). For using of these clinical materials for research purposes, prior patients’ written informed consents and approval from the Institutional Research Ethics Committee of Sun Yat-sen University Cancer Center were obtained. Total RNA and protein were extracted from those tissues and detected by qRT-PCR and western blot, respectively.

### Statistical analysis

Statistical analysis was determined using Fisher’s exact probability for Fig. 1a, Spearman test for Fig. 1b,c, Welch’s two-tailed *t*-test for Fig. 5e and Supplementary Fig. 5b and the Student’s *t*-test for other data. Data were presented as the mean (±s.d.) or mean (±s.e.m.) of at least three independent experiments. *P*<0.05 was considered significantly different.

### Data availability

The RNA-seq and ChIP-seq data reported in this paper have been deposited in the NCBI GEO, under the accession code GSE86504. All other remaining data are available within the Article and Supplementary Files, or available from the authors upon request.

## Additional information

**How to cite this article:** Wu, G. *et al*. Menin enhances c-Myc-mediated transcription to promote cancer progression. *Nat. Commun.* **8**, 15278 doi: 10.1038/ncomms15278 (2017).

**Publisher’s note:** Springer Nature remains neutral with regard to jurisdictional claims in published maps and institutional affiliations.

## Supplementary Material

Supplementary Information Supplementary Figures, Supplementary Tables and Supplementary Reference
