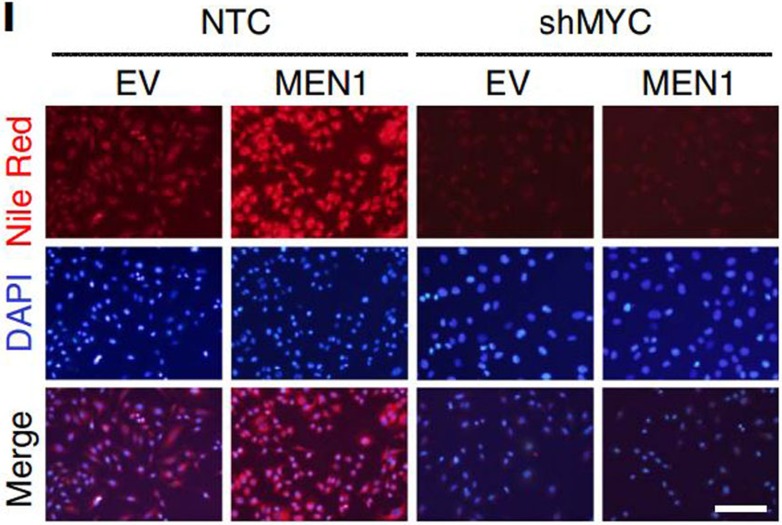# Author Correction: Menin enhances c-Myc-mediated transcription to promote cancer progression

**DOI:** 10.1038/ncomms16195

**Published:** 2018-03-30

**Authors:** Gongwei Wu, Mengqiu Yuan, Shengqi Shen, Xiaoyu Ma, Jingwen Fang, Lianbang Zhu, Linchong Sun, Zhaoji Liu, Xiaoping He, De Huang, Tingting Li, Chenchen Li, Jun Wu, Xin Hu, Zhaoyong Li, Libing Song, Kun Qu, Huafeng Zhang, Ping Gao

Nature Communications 8: Article number: 15278; DOI: 10.1038/ncomms15278 (2017); Published 05 05 2017; Updated 03 30 2018

The originally published version of this Article contained errors in Fig. 6. In panel l, the Nile Red and Merge images of cells treated with shMYC were inadvertently duplicated from the equivalent images of cells treated with MEN1-sh1 and MEN-sh2 in panel n of the same figure. These errors have now been corrected in the PDF and HTML versions of the Article. For comparison, the original, incorrect version of Figure 6l is presented below as Fig. 1.

## Figures and Tables

**Figure 1:**