# Supplementary material for: Menin enhances c-Myc-mediated transcription to promote cancer progression
Source: Nat Commun. 2017 May 5;8:15278. doi: 10.1038/ncomms15278 (PMC5424160; doi:10.1038/ncomms15278)
Supplement: Supplementary Information — Supplementary Figures, Supplementary Tables and Supplementary Reference [file ncomms15278-s1.pdf]

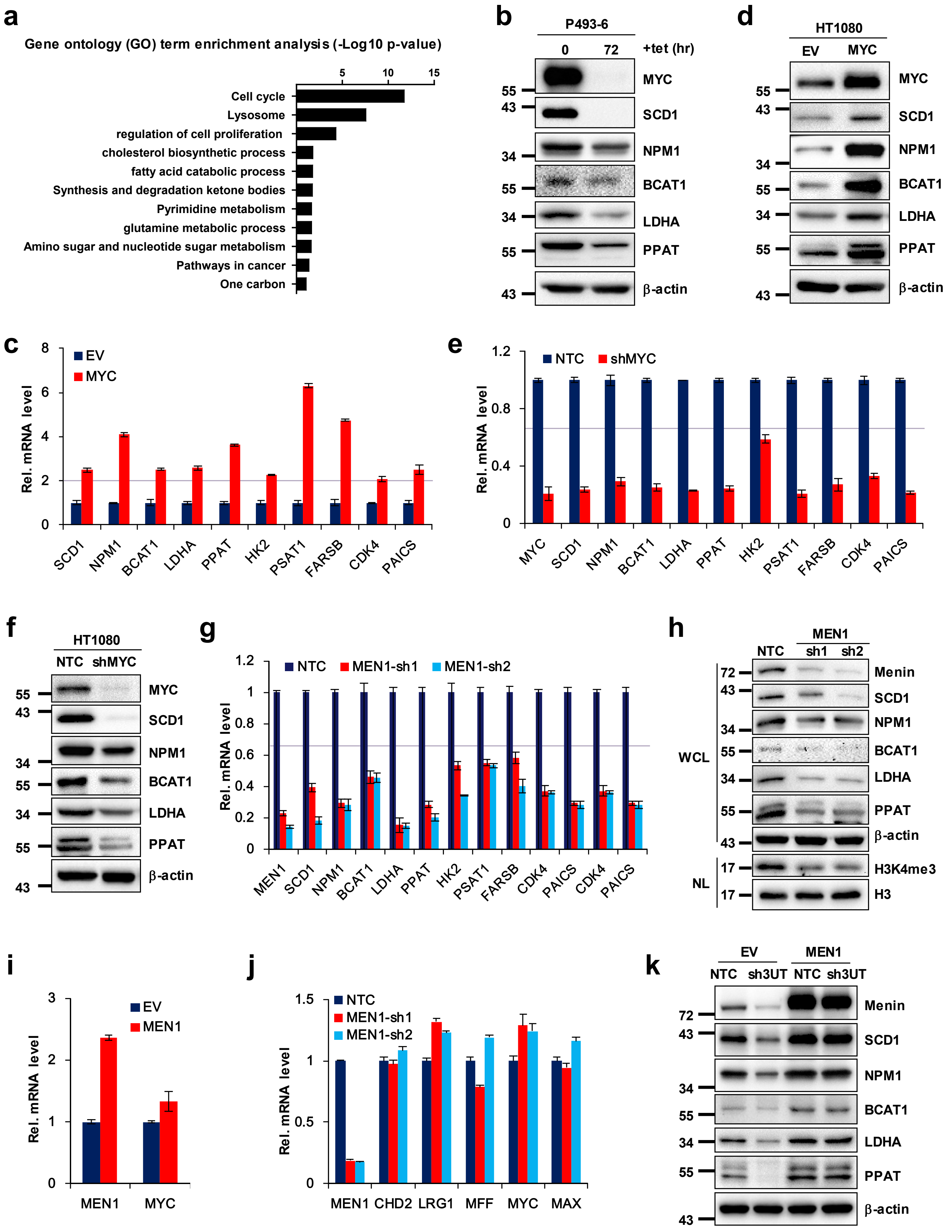
a
Gene ontology (GO) term enrichment analysis (-Log10 p-value)
5 10 15
Cell cycle
Lysosome
regulation of cell proliferation
cholesterol biosynthetic process
fatty acid catabolic process
Synthesis and degradation ketone bodies
Pyrimidine metabolism
glutamine metabolic process
Amino sugar and nucleotide sugar metabolism
Pathways in cancer
One carbon
b
P493-6
0 72 +tet (hr)
55 43 34 55 34 55 43
MYC
SCD1
NPM1
BCAT1
LDHA
PPAT
β-actin
d
HT1080
EV MYC
55 43 34 55 34 55 43
MYC
SCD1
NPM1
BCAT1
LDHA
PPAT
β-actin
c
EV
MYC
Rel. mRNA level
0 2 4 6 8
SCD1 NPM1 BCAT1 LDHA PPAT HK2 PSAT1 FARSB CDK4 PAICS
e
NTC
shMYC
Rel. mRNA level
0 0.4 0.8 1.2
MYC SCD1 NPM1 BCAT1 LDHA PPAT HK2 PSAT1 FARSB CDK4 PAICS
f
HT1080
NTC shMYC
55 43 34 55 34 55 43
MYC
SCD1
NPM1
BCAT1
LDHA
PPAT
β-actin
g
NTC
MEN1-sh1
MEN1-sh2
Rel. mRNA level
0 0.2 0.4 0.6 0.8 1 1.2
MEN1 SCD1 NPM1 BCAT1 LDHA PPAT HK2 PSAT1 FARSB CDK4 PAICS CDK4 PAICS
h
MEN1
NTC sh1 sh2
WCL
NL
72 43 34 55 34 55 43 17 17
Menin
SCD1
NPM1
BCAT1
LDHA
PPAT
β-actin
H3K4me3
H3
i
EV
MEN1
Rel. mRNA level
0 1 2 3
MEN1 MYC
j
NTC
MEN1-sh1
MEN1-sh2
Rel. mRNA level
0 0.5 1 1.5
MEN1 CHD2 LRG1 MFF MYC MAX
k
EV MEN1
NTC sh3UT NTC sh3UT
72 43 34 55 34 55 43
Menin
SCD1
NPM1
BCAT1
LDHA
PPAT
β-actin

Supplementary Figure 1. Analyses of RNA-seq data and detection of MYC and MYC target genes. (**a**) Gene ontology (GO) term enrichment analysis using DAVID for the genes significantly regulated by both MYC and Menin. (**b**) Western blotting assay for MYC and MYC target genes in P493-6 B cells treated with tet for 0 hr or 72 hr. (**c, e**) qRT-PCR assay for the mRNA expression of *MYC* and MYC target genes in HT1080 cells expressing EV or MYC (**c**), and NTC or sh*MYC* (**e**). (**d, f**) Western blotting assay for MYC and MYC target genes in HT1080 cells stably expressing EV or MYC (**d**), and NTC or sh*MYC* (**f**). (**g**) qRT-PCR assay for *MEN1* and MYC target genes in HepG2 cells expressing NTC or sh*MEN1*. (**h**) Western blotting assay for protein levels of Menin, H3K4me3 and MYC target genes in HepG2 cells expressing NTC or sh*MEN1*. β-actin and H3 serve as loading controls. WCL: whole cell lysate; NL: nuclear lysate. (**i, j**) qRT-PCR assay for the mRNA expression of *MEN1* and *MYC* in HT1080 cells expressing EV or Menin (**i**) and the mRNA expression of MYC and MAX as well as three negative control genes that were not regulated by MYC and Menin in HT1080 cells expressing NTC or sh*MEN1* (**j**). (**k**) HT1080 cells expressing NTC or shRNA targeting 3′ UTR of *MEN1* were further infected with viruses expressing EV or Menin. Protein levels of Menin and MYC target genes were detected by Western blotting. Result is representative of three independent experiments. Error bars correspond to s.d. β-actin serves as loading control.

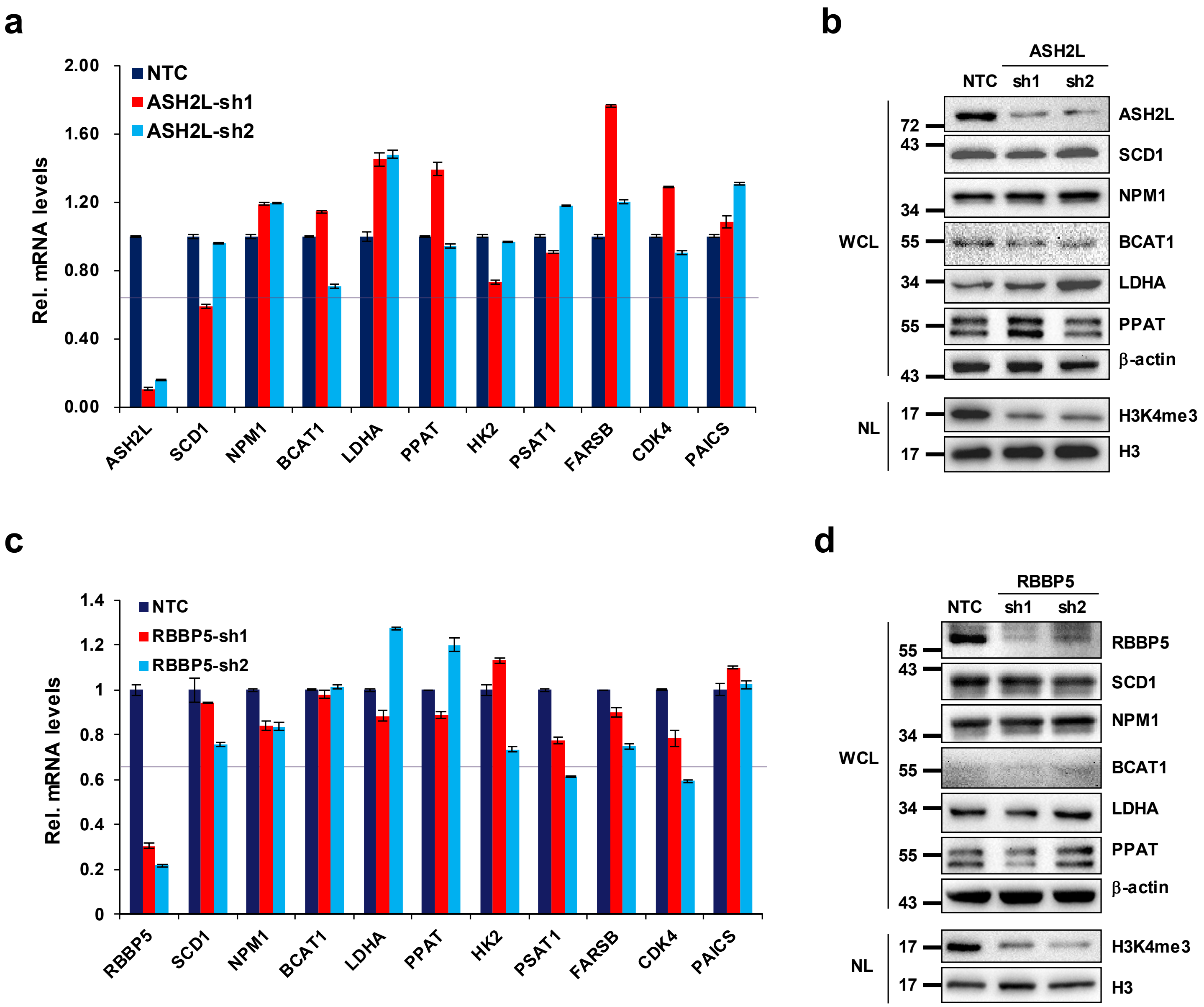

Supplementary Figure 2. H3K4me3 is not involved in the enhanced transcription of MYC target genes by Menin. (**a, c**) qRT-PCR assay for the mRNA expression of MYC target genes in HT1080 cells infected with viruses expressing NTC or sh*ASH2L* (**a**), and NTC or sh*RBBP5* (**c**). Data were presented as mean (±SD). (**b, d**) Western blotting assay for MYC target genes and H3K4me3 levels in HT1080 cells infected with viruses expressing NTC or sh*ASH2L* (**b**), and NTC or sh*RBBP5* (**d**). β-actin and H3 serve as loading controls. WCL: whole cell lysate; NL: nuclear lysate.

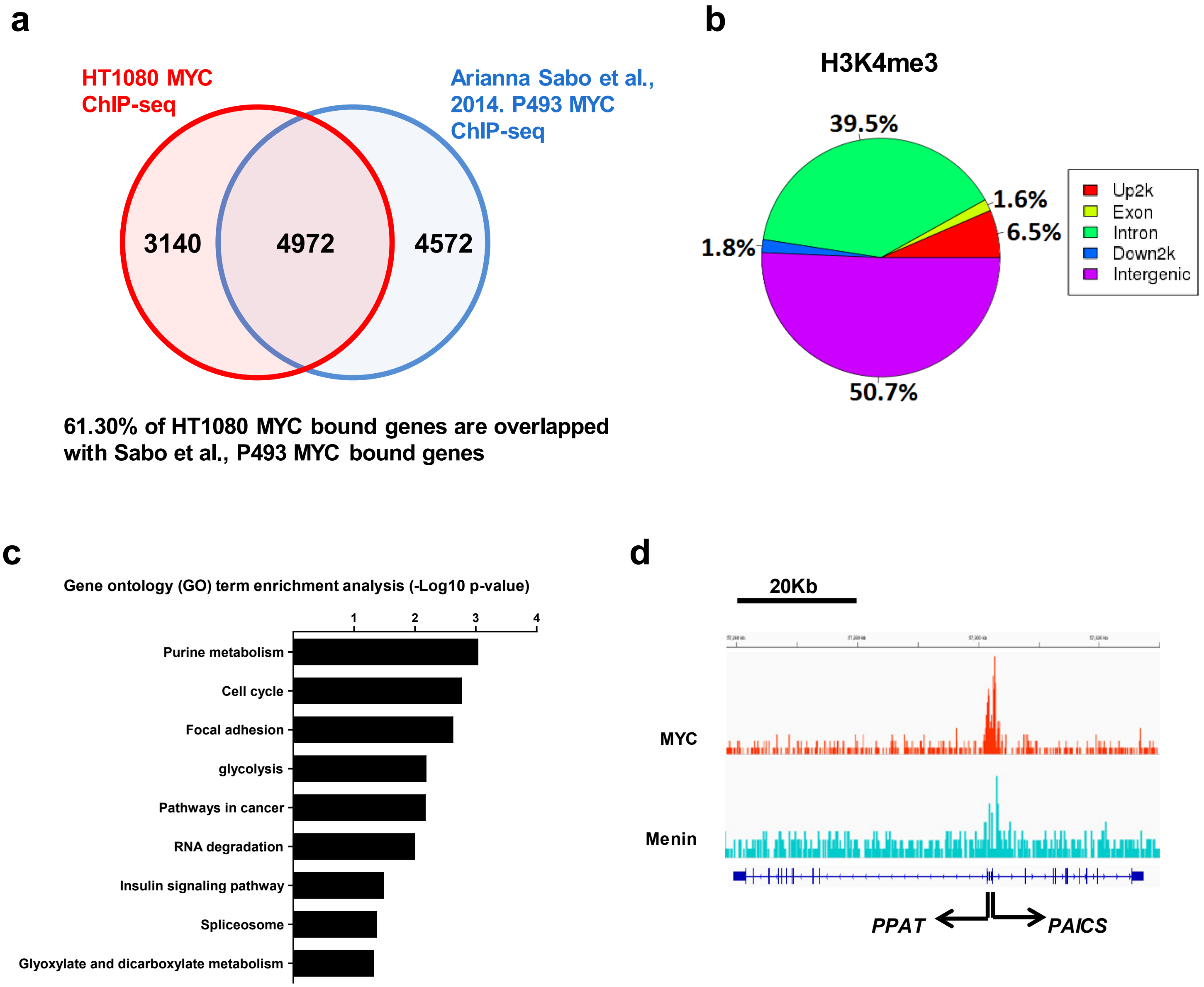

Supplementary Figure 3. Analyses of ChIP-seq data. (**a**) Venn diagram of the ChIP-seq data showing that MYC bound genes from our HT1080 MYC ChIP-seq data set are highly overlapped with those from the published P493-6 MYC ChIP-seq data set [1]. (**b**) Pie graph illustrating genomic locations of H3K4me3 peaks. (**c**) Gene ontology (GO) term enrichment analysis using DAVID for MYC and Menin overlapping peaks related genes. (**d**) IGV graph showing gene traces of representative genes.

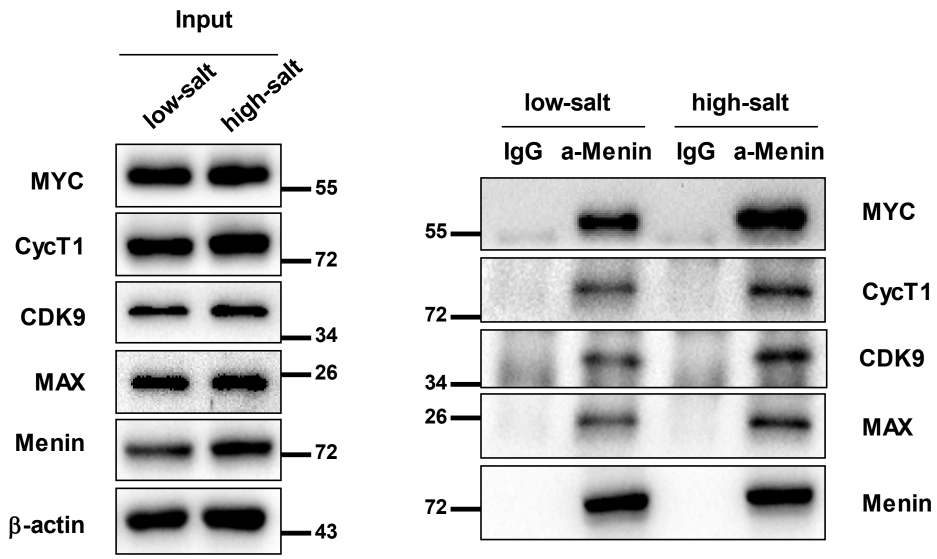

Supplementary Figure 4. Interactions between Menin and MYC, Menin and P-TEFb. Interactions between endogenous Menin and MYC, Menin and P-TEFb in cells lysed with low-salt buffer (150 mM) or with high-salt buffer (300 mM). IP was performed with anti-IgG or anti-Menin antibody, followed by blotting with anti-MYC, anti-MAX or anti-P-TEFb.

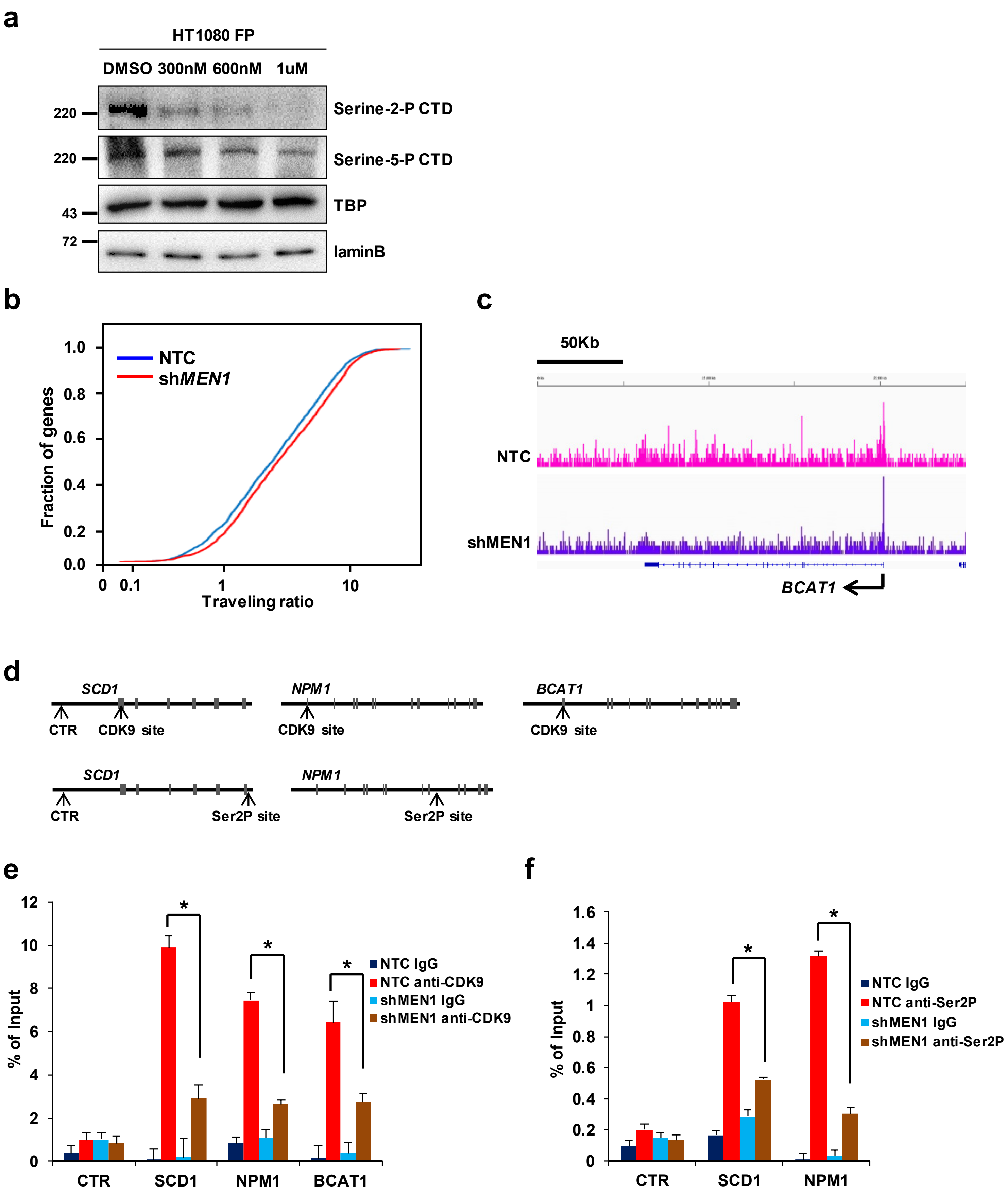

Supplementary Figure 5. Menin is required for CDK9 recruitment and Ser2-phosphorylation at MYC target genes. (**a**) Titration of the flavopiridol (FP) concentration for CDK9 inhibition in HT1080.

HT1080 cells were treated with 300 nM, 600 nM and 1 uM FP for 6 hr. TBP serves as loading control for RNA Pol II. LaminB serves as loading control for total nuclear protein. (**b**) Empirical cumulative distribution plots of RNA Pol II traveling ratio for 3000 transcribed genes which were randomly selected from RNA Pol II ChIP-seq data and P value $<8.16 \times 10^{-8}$ by Welch´s two-tailed t test. (**c**) IGV graph showing gene traces of RNA Pol II occupancy at *BCAT1* gene. (**d**) Abridged general view of primer amplification sites for CDK9 and Ser2P. (**e, f**) ChIP experiments were performed in HT1080 cells expressing NTC or sh*MEN1* using IgG or anti-CDK9 antibody **(e)** or anti-Ser2P antibody **(f)**. The occupancy of CDK9 or Ser2P was determined by qRT-PCR. Data were presented as mean (±SD). * P<0.05 as compared between indicated groups.

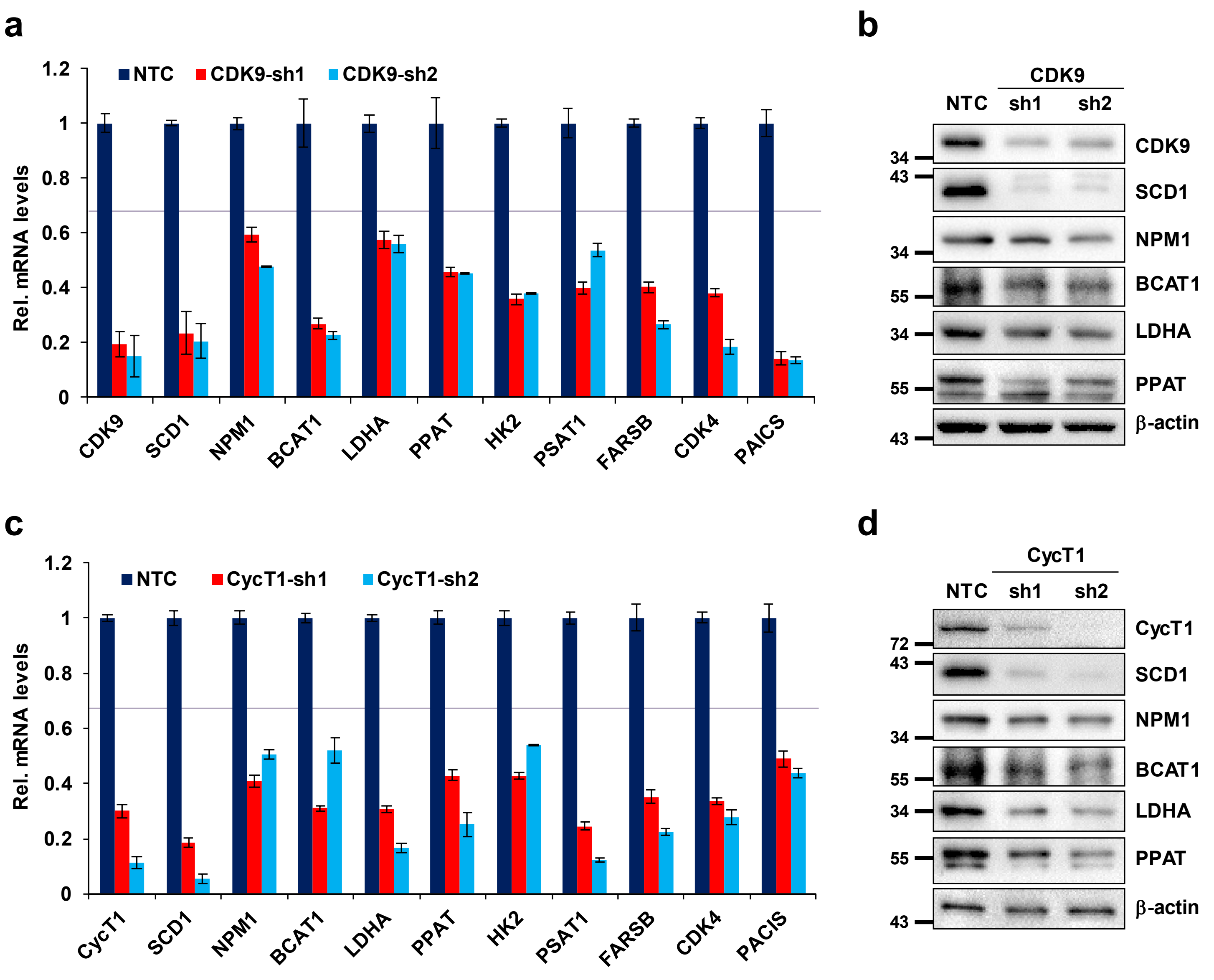

Supplementary Figure 6. P-TEFb regulates MYC target gene expression. (**a, c**) qRT-PCR assay for the mRNA expression levels of MYC target genes in HT1080 cells expressing NTC or sh*CDK9* (**a**), and NTC or sh*CycT1* (**c**). Data were presented as mean (±SD) of three independent experiments. (**b, d**) Western blotting assay for proteins from HT1080 cells expressing NTC or sh*CDK9* (**b**), and NTC or sh*CycT1* (**d**). β-actin serves as loading control.

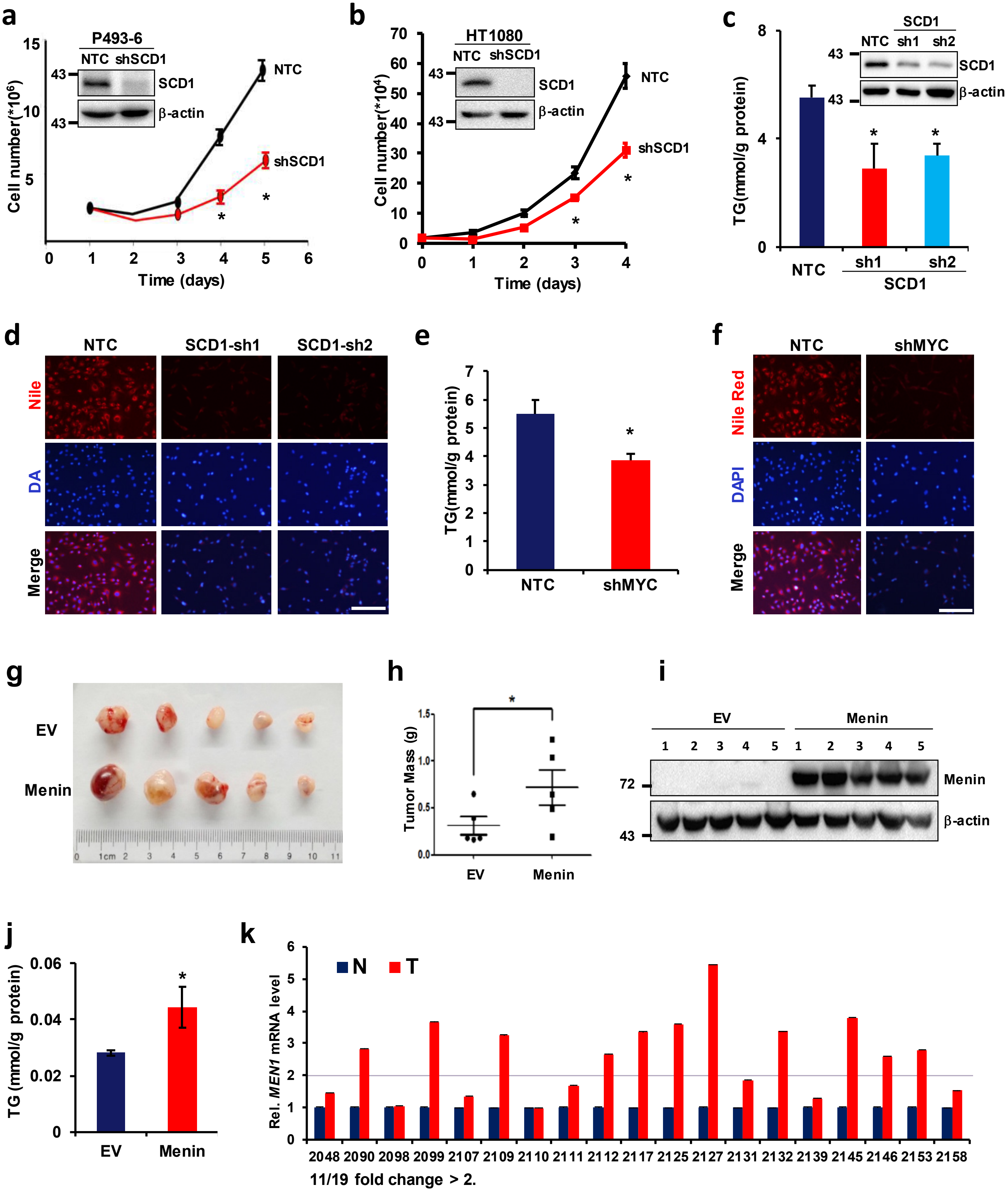

Supplementary Figure 7. Menin is critical for MYC-mediated cell metabolism and cancer progression.

(**a, b**) Cell growth curves of P493-6 cells (**a**) and HT1080 cells (**b**) transfected with NTC or sh*SCD1*. The cell growth was determined by trypan blue counting. Data were presented as mean (±SD). * P<0.05 as compared to NTC group. (**c, e**) TG concentrations were measured using cell lysates from HT1080 cells expressing NTC or sh*SCD1* (**c**), and NTC or sh*MYC* (**e**). The values were normalized to cellular protein. Data were presented as mean (±SD) of three independent experiments. Western blot showing the knockdown efficiency. * P<0.05 as compared to NTC group. (**d, f**) The lipid droplets were determined in HT1080 cells expressing NTC or sh*SCD1* (**d**), and NTC or sh*MYC* (**f**) by Nile Red and DAPI staining. Scale bars, 100 uM. (**g-i**) HT1080 cells stably expressing EV or Menin were injected subcutaneously into nude mice (n=5 for each group). Picture showing the tumors collected at the end of the experiment (day 20) (**g**). Tumor mass was measured at the end of the experiment (**h**). Overexpression of Menin in tumors were confirmed by Western blot (**i**). β-actin serves as loading control. Data were presented as mean (±SEM). * P<0.05 as compared to EV group. (**j**) TG concentrations in the lysates from extracted tumors were measured and the values were normalized to protein. Data were presented as mean (±SD). * P<0.05 as compared to NTC control group. (**k**) mRNA expression of *MEN1* were determined by qRT-PCR in 19 pairs of clinically matched tumor adjacent non-cancerous liver tissues (normal) and human hepatocellular carcinoma (HCC) tissues (tumor). mRNA levels were normalized to 18S rRNA.

Fig. 1

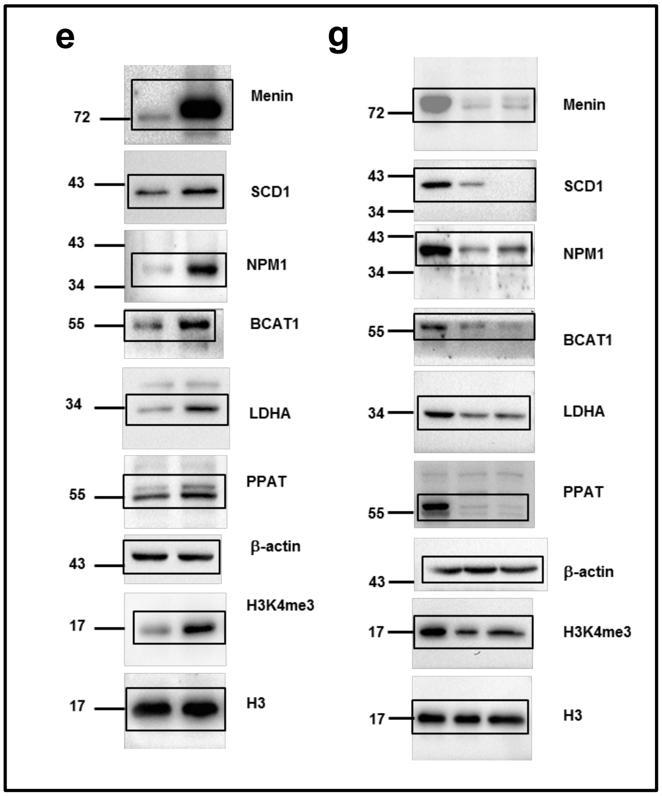

Fig. 2

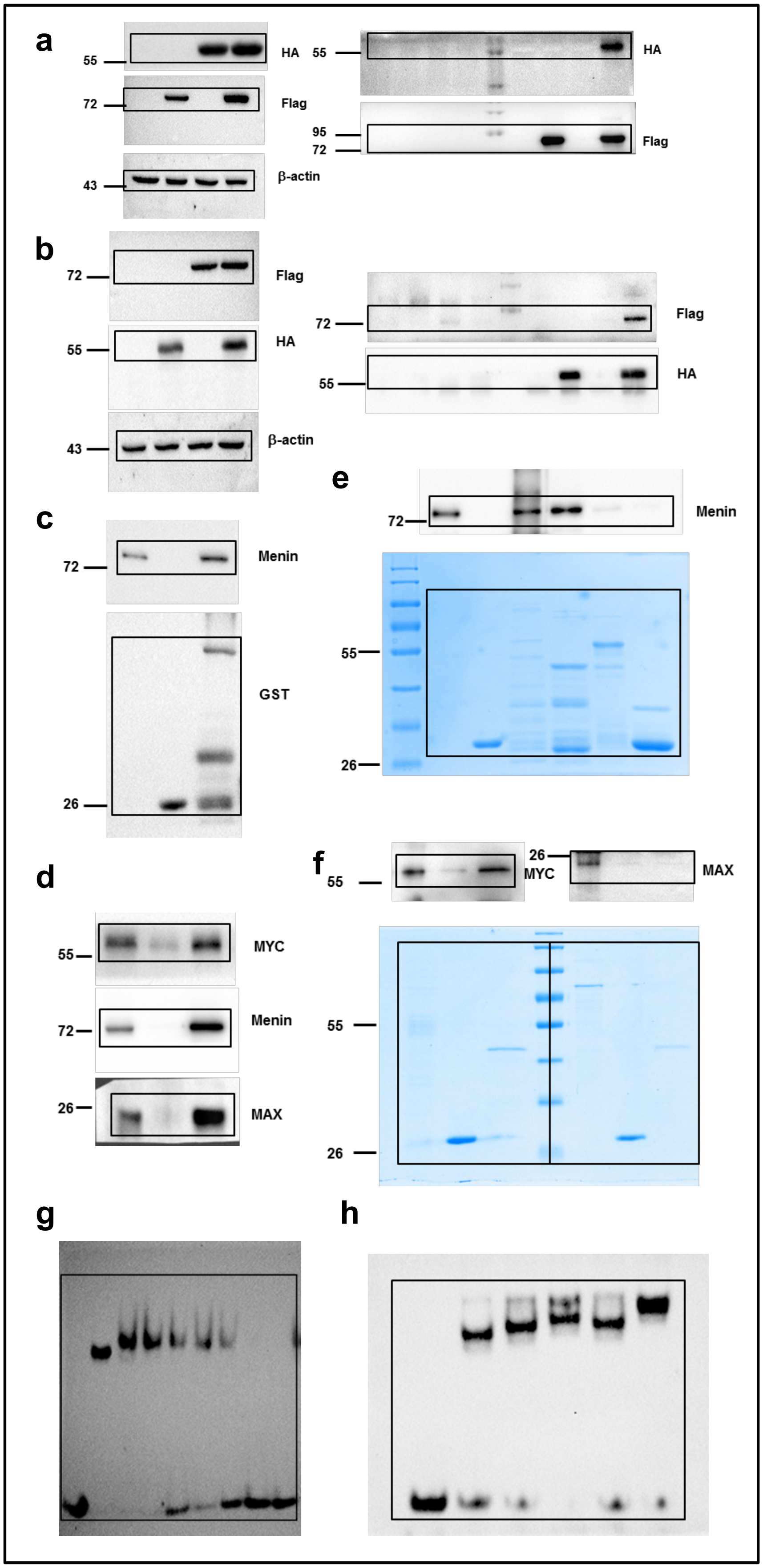

Fig. 3

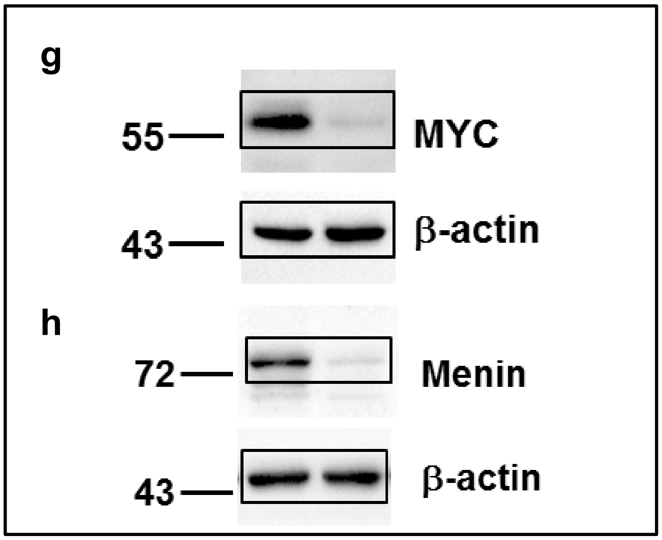

Supplementary Figure 8. The uncropped scans of immunoblots.

Fig. 4

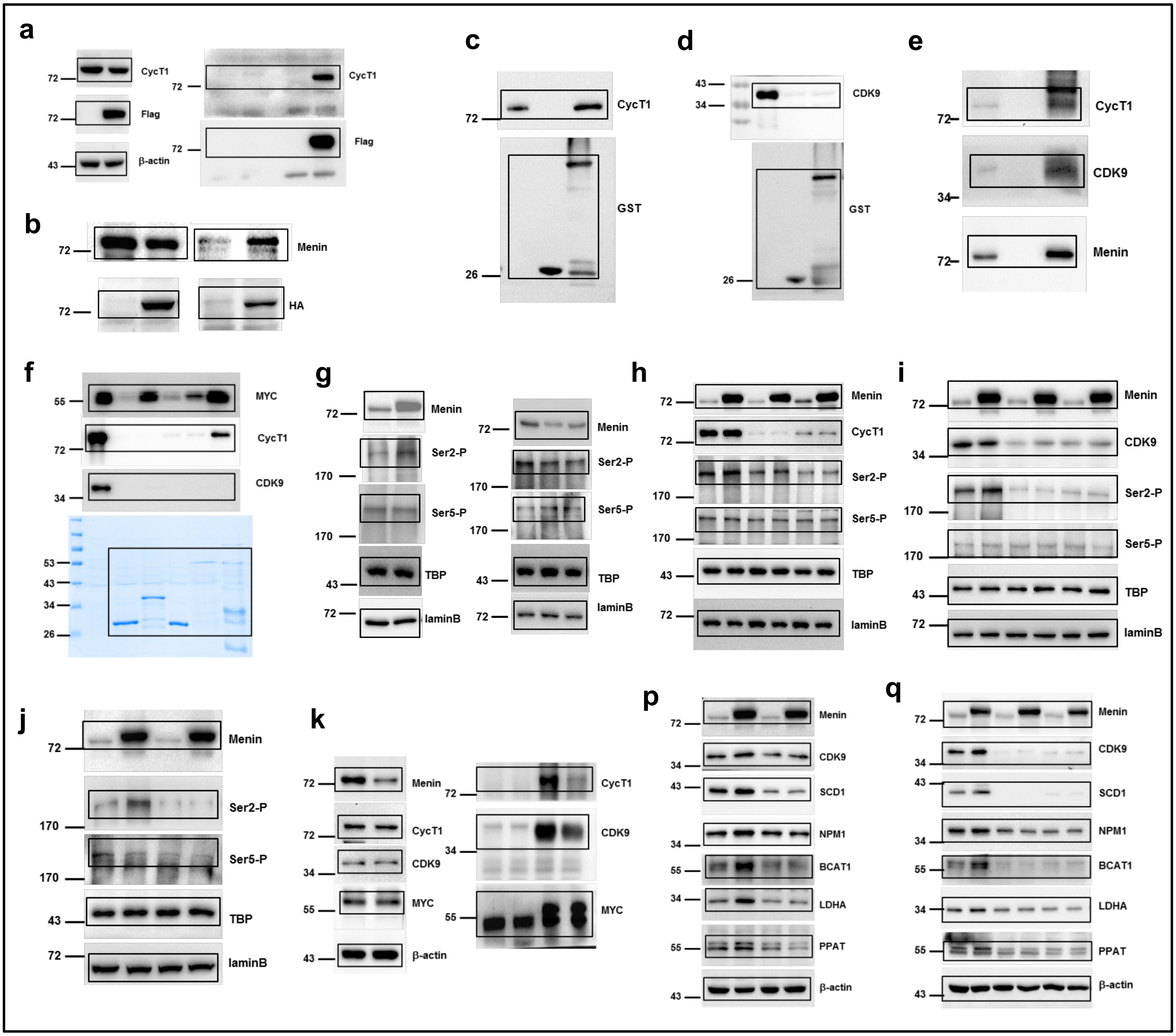

Fig. 5

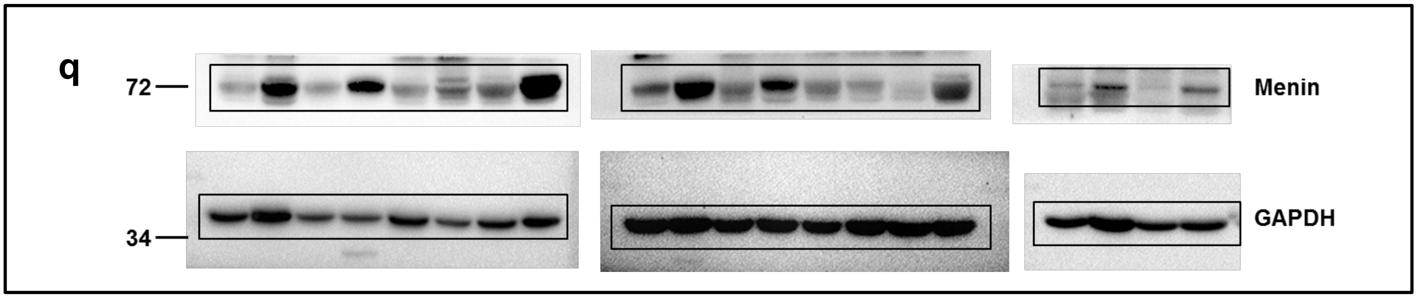

Supplementary Figure 8. The uncropped scans of immunoblots (continued).

Fig. S1

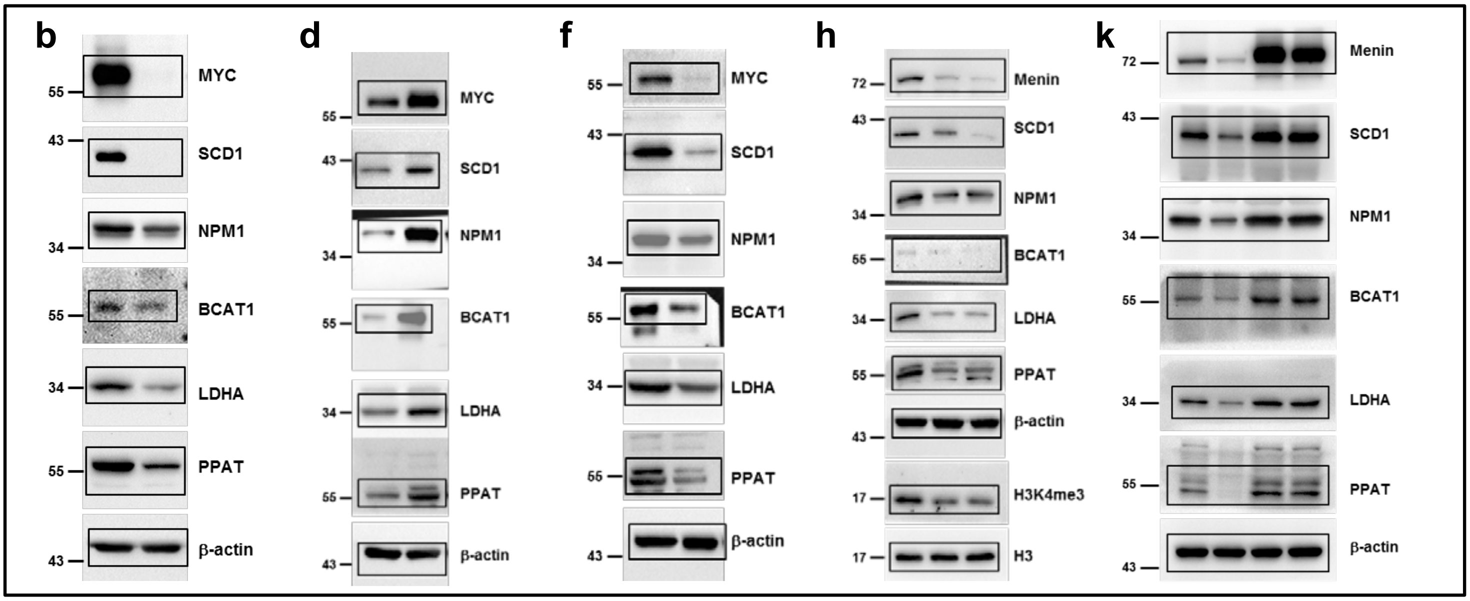

Fig. S2

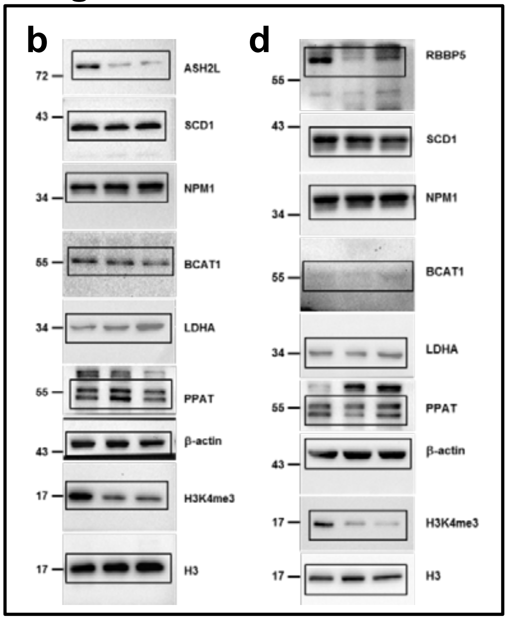

Fig. S5

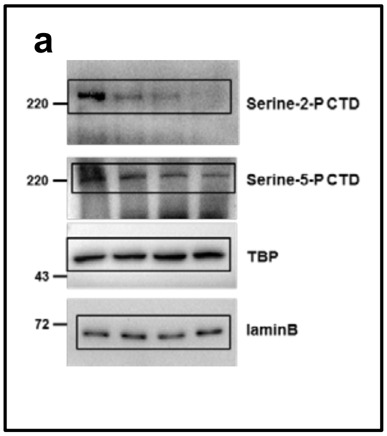

Fig. S6

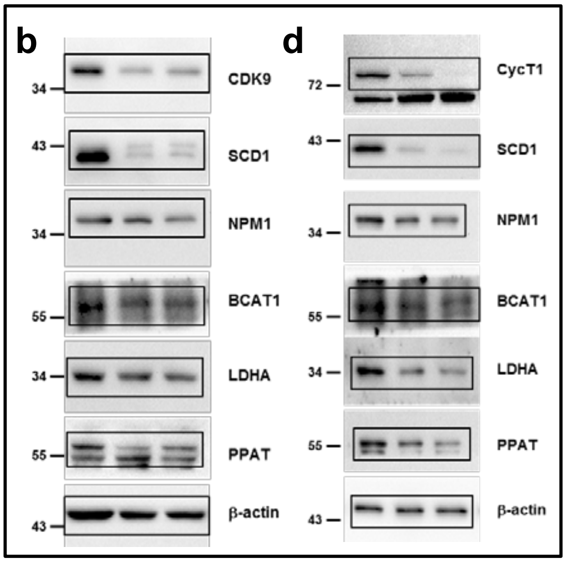

Fig. S7

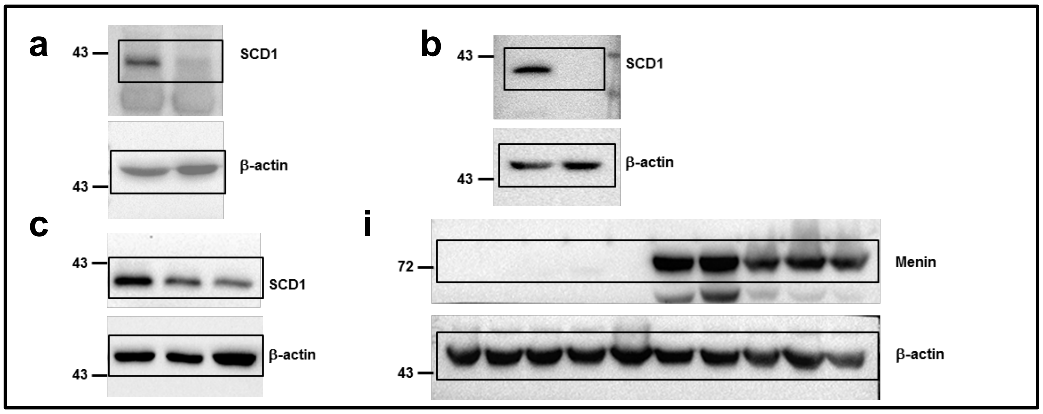

Supplementary Figure 8. The uncropped scans of immunoblots (continued).

Supplementary Table 1. shRNAs used in this paper

shRNA	shRNA ID	Sequence
Non-Target shRNA Control	SHC002	CCGGCAACAAGATGAAGAGCACCAACTCGAGTTGGTGCTCTTCATCTTGTTGTTTTT
MYC	TRCN0000039638	CCGGCCATAATGTAAACTGCCTCAACTCGAGTTGAGGCAGTTTACATTATGGTTTTTG
MEN1-sh1	TRCN0000040139	CCGGGCTGTACCTGAAAGGATCATACTCGAGTATGATCCTTTCAGGTACAGCTTTTTG
MEN1-sh2	TRCN0000338331	CCGGCTGTACCTGAAAGGATCATACCTCGAGGTATGATCCTTTCAGGTACAGTTTTTG
sh3UTR	TRCN0000009849	CCGGAATTCCATATCTTGCGAATCCTCGAGGATTCGCAAGATATGGAATTCTTTTTG
SCD-sh1	TRCN0000056613	CCGGCTACGGCTCTTTCTGATCATTCTCGAGAATGATCAGAAAGAGCCGTAGTTTTTG
SCD-sh2	TRCN0000056616	CCGGGCACATCAACTTCACCACATTCTCGAGAATGTGGTGAAGTTGATGTGCTTTTTG
ASH2L-sh1	TRCN0000019275	CCGGCCGAAGACAATGTTCTCCAAACTCGAGTTTGGAGAACATTGTCTTCGGTTTTT
ASH2L-sh2	TRCN0000019277	CCGGCCCGTTTAACAAAGATGGCTACTCGAGTAGCCATCTTTGTTAAACGGGTTTTT
RBBP5-sh1	TRCN0000165444	CCGGGCCACCTAAGAAGAAACCCAACTCGAGTTGGGTTTCTTCTTAGGTGGCTTTTTTG
RBBP5-sh2	TRCN0000159189	CCGGGCAGATCGAATAATCAGAGTTCTCGAGAACTCTGATTATTCGATCTGCTTTTTTG
CDK9-sh1	TRCN0000000497	CCGGCTACTACATCCACAGAAACAACTCGAGTTGTTTCTGTGGATGTAGTAGTTTTT
CDK9-sh2	TRCN0000199857	CCGGGCTGGGCTGTTGAGCAATGTTCTCGAGAACATTGCTCAACAGCCCAGCTTTTTTG
Cyclin T1-sh1	TRCN0000013674	CCGGGCCAATGTGAAGTCACAATATCTCGAGATATTGTGACTTCACATTGGCTTTTT
Cyclin T1-sh2	TRCN0000013677	CCGGCGGTGGTATTTCACTCGAGAACTCGAGTTCTCGAGTGAAATACCACCGTTTTT

Supplementary Table 2. qRT-PCR primers used in this paper

Primers used for mRNA qRT-PCR			
**Gene name**	**GenBank Accession**	**sequence of forward primer**	**sequence of reverse primer**
18s		CGGCGACGACCCATTCGAAC	GAATCGAACCCTGATTCCCCGTC
HK2	NM_000189	CCAGTTCATTCACATCATCAG	CTTACACGAGGTCACATAGC
ASH2L	NM_004674	ATGGCAGTCCCGAACACAG	GGTTGTCATGCACTCCCAGTAT
SCD1	NM_005063	AGAATGGAGGAGATAAGT	TAGCAGAGACATAAGGAT
MEN1	NM_000244	CCATATCTTGCGAATCCA	TACGAAGGAGAGGAAACT
CDK4	NM_001262	CTGGTGTTTGAGCATGTAGACC	GATCCTTGATCGTTTCGGCTG
LDHA	NM_001165416	GGCTACAACAGGATTCTA	TTACAAACCATTCTTATTTCTAAC
MYC	NM_002467	AACGATTCCTTCTAACAG	GGCTAAATCTTTCAGTCT
CDK9	NM_001261	TTGCGGGAGATCAAGATCCTT	TACCCTTGCAGCGGTTATAGG
Cyclin T1	NM_001240	CAGTGTTACAAAGAGCCGAGAG	GAGTGCTTGTGTGAGTGGTG
PAICS	NM_001079525	ACAAACAGTCTTATCGGGACCT	CTGCAACCCACTCAAAGTTTTTC
BCAT1	NM_005504	AGCCCTGCTCTTTGTACTCTT	CCAGGCTCTTACATACTTGGGA
PSAT1	NM_021154	CGGTCCTGGAATACAAGGTG	AACCAAGCCCATGACGTAGA
RBBP5	NM_005057	CATCTTTTGATAGGCGAGGGG	GTTCCAGTTGTCACTCTGAAGG
FARSB	NM_005687	ACTGTCAGCGTGAAGCGTG	CTGCTGCCTTTACATTACCTTGT
PPAT	NM_002703	AATTGTCAGCCCTTCGTTGTT	CCTTAATCGAGCAGCATTTACCA
NPM1	NM_199185	AGCGCCAGTGAAGAAAGGAC	GCCTCTTGGTCAGTCATCCG

Primers used for ChIP qRT-PCR		
**Primer name**	**sequence of forward primer**	**sequence of reverse primer**
SCD1-Ebox	GAAGGGTCCGTACTGTCCACC	AGTCAGGAGAAGAGACACGTGGA
SCD1-CTR	TCTAGCTGTTTCCCTAGGAATGAA	GCGTCCCCATATCTTCCTCTG
NPM1-Ebox	CTCGTGAGCCAGGGATGCT	GCTGCCATCACAGTACATGCT
NPM1-CTR	GTGCCAGTTCTTGGGTTTGT	ATGGAAACACTCCCCGAATA
BCAT1-Ebox	GCCTTAGTGTCTTCCTGCTGA	AGCAAGACCTGGGGCAGT
BCAT1-CTR	TGGGTGGGTGCCTTTGATATG	TTTCAACACTAGGCTGGGCTTA
CDKN2C	CTCCACAACCGTCTTAAATAACAAACC	GCGGGCTTGAGTCTGTGA
CDK9/Ser2P-CTR	CAGCTTCAAGGGTAAGTGGTGA	AGAAGTGGGTGAGGCTTTTAGT
SCD1-CDK9	ACGAGCCGGAGTTTACAGAAG	CAAAGTGCGAGGAGTTGACTG
NPM1-CDK9	ATATAAGCGCGGGGAGCCTG	GAGAGAAGGCGGACGGAGAT
BCAT1-CDK9	TGATGCAATCCGCTAGGTCG	CAGATCCCAAGGGTCGTAGC
NPM1-Ser2P	CACATTAACACCAGCTGGCAAC	ATGTGACCGACCACGTGACA
BCAT1-Ser2P	TAGGCCTTTATAGAACCCCTGTA	ACCTACAACACACAACAAGCTGA

Supplementary Reference

1 Sabo, A. *et al*. Selective transcriptional regulation by Myc in cellular growth control and lymphomagenesis. *Nature* **511**, 488-492, doi:10.1038/nature13537 (2014).
